# What public health challenges and unmet medical needs would benefit from interdisciplinary collaboration in the EU? A survey and multi-stakeholder debate

**DOI:** 10.3389/fpubh.2024.1417684

**Published:** 2024-07-22

**Authors:** Francesca Pistollato, Gregor Burkhart, Pierre Deceuninck, Camilla Bernasconi, Sergio Di Virgilio, Luca Emili, Anne-Charlotte Fauvel, Luisa Ferreira Bastos, Annalisa Gastaldello, Chiara Gerardi, Jens K. Habermann, Ioan Hanes, Christina Kyriakopoulou, Uma Lanka, Paolo Lauriola, Hugh Laverty, Benoit G. C. Maisonneuve, Milena Mennecozzi, Francesco Pappalardo, Roberta Pastorino, Vilma Radvilaite, Erwin L. Roggen, Helder Constantino

**Affiliations:** ^1^Research and Toxicology, Humane Society International, Brussels, Belgium; ^2^European Monitoring Centre for Drugs and Drug Addiction (EMCDDA), Lisbon, Portugal; ^3^European Commission, Joint Research Centre (JRC), Ispra, Italy; ^4^European Commission, Research and Innovation (R&I), Brussels, Belgium; ^5^InSilicoTrials Technologies, Milan, Italy; ^6^European Infrastructure for Translational Medicine, Amsterdam, Netherlands; ^7^Eurogroup for Animals, Brussels, Belgium; ^8^Center for Health Regulatory Policies, Mario Negri Institute for Pharmacological Research IRCCS, Milan, Italy; ^9^BBMRI-ERIC, Biobanking and Biomolecular Resources Research Infrastructure Consortium, Graz, Austria; ^10^European Lifestyle Medicine Organization, Geneva, Switzerland; ^11^Research and Toxicology, Humane Society International, London, United Kingdom; ^12^International Society of Doctors for the Environment, Modena, Italy; ^13^Innovative Health Initiative, Brussels, Belgium; ^14^NETRI – Digitizing Human Biology, Lyon, France; ^15^Department of Drug and Health Sciences, University of Catania, Catania, Italy; ^16^Section of Hygiene, Department of Life Sciences and Public Health, Università Cattolica del Sacro Cuore, Rome, Italy; ^17^European Innovation Council, Brussels, Belgium; ^18^ToxGenSolutions and 3Rs Management & Consulting ApS, Maastricht, Netherlands

**Keywords:** public health, biomedical research, patient-centric research, societal impact, policy, translatability, funding, research innovative methodologies

## Abstract

In the past decade, significant European calls for research proposals have supported translational collaborative research on non-communicable and infectious diseases within the biomedical life sciences by bringing together interdisciplinary and multinational consortia. This research has advanced our understanding of disease pathophysiology, marking considerable scientific progress. Yet, it is crucial to retrospectively evaluate these efforts’ societal impact. Research proposals should be thoughtfully designed to ensure that the research findings can be effectively translated into actionable policies. In addition, the choice of scientific methods plays a pivotal role in shaping the societal impact of research discoveries. Understanding the factors responsible for current unmet public health issues and medical needs is crucial for crafting innovative strategies for research policy interventions. A multistakeholder survey and a roundtable helped identify potential needs for consideration in the EU research and policy agenda. Based on survey findings, mental health disorders, metabolic syndrome, cancer, antimicrobial resistance, environmental pollution, and cardiovascular diseases were considered the public health challenges deserving prioritisation. In addition, early diagnosis, primary prevention, the impact of environmental pollution on disease onset and personalised medicine approaches were the most selected unmet medical needs. Survey findings enabled the formulation of some research-policies interventions (RPIs), which were further discussed during a multistakeholder online roundtable. The discussion underscored recent EU-level activities aligned with the survey-derived RPIs and facilitated an exchange of perspectives on public health and biomedical research topics ripe for interdisciplinary collaboration and warranting attention within the EU’s research and policy agenda. Actionable recommendations aimed at facilitating the translation of knowledge into transformative, science-based policies are also provided.

## 1 Introduction

EU-funded research has played a pivotal role in driving crucial scientific progress and breakthroughs, enriching our understanding of the pathophysiology of human diseases [e.g., (1, 2)]. These research activities have facilitated the discovery of novel molecular and cellular mechanisms responsible for the onset and progression of infectious diseases as well as common non-communicable diseases (NCDs) (3–6). However, even with significant research achievements, the burden of diseases like dementia or cancer on patients and society has not exhibited a significant decrease over time (7, 8). NCDs have been responsible for 90% of all deaths in the EU in 2021 (9), killing about 41 million people each year globally, according to a most recent 2023 WHO report (10). In 2016, NCDs made up the most significant portion of the national healthcare expenses, totaling 115 billion euros annually in the EU, which amounts to 0.8% of the GDP (11). These diseases also result in other societal costs, such as decreased productivity and workforce implications (12).

When considering drug development, the overall failure rate remains exceptionally high, with 90–95% of tested drugs not reaching regulatory approval (13, 14). Issues related to preclinical experimentation design, including the choice of inadequately predictive *in vitro* and *in vivo* models, the improper selection of drug targets, neglecting pharmacodynamic and pharmacokinetic properties of new compounds, or the inaccurate selection of participants for clinical trials (i.e., not accounting for disease heterogeneity), are seen as plausible reasons contributing to the clinical failures encountered in drug development (3, 15–17).

Moreover, promoting primary prevention is crucial, given that the majority, if not all, of chronic NCDs can be significantly averted through this type of interventions. Various factors, including diet, physical activity, tobacco and alcohol misuse, etc., have an impact on the onset of these health conditions (18). In particular, the diet also plays a role in influencing immune response and susceptibility to infectious diseases (see, e.g., the Special Issue on this topic published in *Nutrients*). Encouraging initiatives in primary prevention can have a substantial impact on public health. Notwithstanding, while chronic diseases, on average, account for up to 80% of EU healthcare costs, preventive healthcare in the EU accounted for 0.37% of GDP in 2020 (19).

In addition, the impact of environmental pollutants on the onset of many NCDs and the risk of developmental disorders has been confirmed by multiple studies [reviewed in (20)]. The repercussions of exposure may extend systemically, causing harm to the liver, kidneys, nervous system, blood, cardiovascular system, immune system, metabolism or reproductive system. Specific pollutants can also induce carcinogenic, teratogenic, and mutagenic effects (21). Investigating the impact of the environment on human health, encompassing research aimed at unravelling the intricate connections between environmental exposures, human biology, genetics, diseases, and health outcomes, deserves prioritisation, and some recent strategies and policies in the EU are going toward this direction (22).

Beyond generating scientific advancements, publicly funded research, especially in most recent framework programmes, has been focusing on impact-driven approaches to deliver tangible societal benefits. A typical example of outcome-driven research is the European collaboration model for addressing health challenges. In particular, Horizon Europe, one of the largest world programmes, has put a high emphasis on Key Impact Pathways to catalyse effective research at the EU level and beyond (23).

Outputs from funded research should eventually translate into outcomes that enhance public health, reduce disease burden and mortality rates, and contribute to more cost-effective and sustainable healthcare interventions, among other improvements. Ultimately, the goal of such research endeavours should be to positively impact public health and therefore contribute to, e.g., (i) identify disease risk factors and inform policymakers on the need to implement suitable intervention strategies through integrated environmental public approach; (ii) develop and market drugs that are safe and effective for patients; (iii) increase public awareness about disease prevention and healthy and environmentally sustainable lifestyles, disease management and treatment; and (iv) prevent diseases.

However, as mentioned earlier, disease prevalence and incidence rates remain high, which may also be due to lack of or insufficient investment in prevention research, the inefficient implementation of primary prevention strategies, and the high rate of drug attrition. For this reason, it is crucial to define monitoring approaches to assess the impact and effectiveness of funding strategies and the short to long term outcomes generated by funded research. However, assessing the economic and societal impacts of research and comprehending how funded research has effectively delivered solutions for societal needs poses a challenging endeavour (24), while considering that dissemination of research outputs, such as through peer-reviewed publications, is clearly not enough and might not consistently mirror the broader societal impact of the research (25).

Fostering dialogue between researchers (from all sectors both public and private, as well as all disciplines), the public, and policymakers is crucial to effectively address the most urgent public health challenges and unmet medical needs (UMNs). UNM is a crucial concept in the incentivisation and development of new health technologies; it refers to specific medical needs that are not adequately addressed by current medical interventions or treatments, despite advancements in medical research and technology. The identification of a medical need as ‘unmet’ aims to stimulate innovation and prioritise the development of new health technologies in that area (26). In addition, UMN is a dynamic concept, as the definition of ‘need’ continually evolves with advancements in science, technology, data, infrastructure, and collaboration, as well as with the progression of diseases (whether chronic or acute) (27).

Interdisciplinary collaborations can help prioritise main challenges in the research agenda. Some dedicated funding calls have supported research initiatives that are directly relevant to humans and have the potential to drive significant societal impact (28). These should maximise synergies for accelerated scientific advancements by building on already existing research infrastructures and resources, and finding accelerated pathways for their uptake and implementation. Along the same line, sharing knowledge and improving uptake of research and innovation results by the society are key elements of the EC proposal to revitalise the European Research Area (29).

A thorough understanding of the factors responsible for current and emerging public health challenges and the identification of UMNs is crucial for crafting innovative strategies for research and policy interventions. On September 25th 2023, a survey was launched by Humane Society International/Europe (HSI) to gather public health and biomedical research stakeholder representatives’ feedback and opinions on current most urgent public health challenges and UMNs, and possible research policy initiatives deserving prioritisation in the EU (30). Survey fundings and some potential research-policy interventions (RPIs) based on them are reported here in PART I.

In addition, on November 24th 2023, HSI hosted an online roundtable with representatives of research institutions, industry, European Commission (EC) research and funding bodies, public health associations and NGOs to (i) discuss the same topics explored in the survey, (ii) highlight some recent EU initiatives, programs and funding calls that have been launched in recent years and that are directly or indirectly linked to the RPIs derived from survey findings, and (iii) exchange perspectives about public health and biomedical research topics that would benefit from interdisciplinary collaboration. These aspects are discussed in PART II, which showcases also a list of actionable recommendations aimed at facilitating the translation of research into policies.

## 2 Methodological approach

### 2.1 Survey design

The survey was designed by HSI using the EU survey platform https://ec.europa.eu/eusurvey/runner/PublicHealthEU. Survey questions are shown in Supplementary material 1. It comprised an introductory text to present the overall goals of the survey, some questions aimed at gathering information about survey participants (‘General Details’ section), followed by seven multiple-choice questions aimed at exploring:

- What public health challenges and UMNs deserve prioritisation in the EU research/policy agenda;
- What factors may contribute to prevalence and incidence of diseases (e.g., NCDs);
- What research activities deserve more resources to face public health challenges and UMNs;
- What possible strategies could be considered to make EU-funded research more translational and impactful;
- What policy interventions could be deemed suitable to tackle emerging/unsolved public health challenges.

The public health challenges listed as possible options under Q1, have been chosen based on the European Commission’s focus on cancer (31), mental health disorders (32), and NCDs (12), as well as a recent analysis identifying public health challenges that require coordinated interventions and solutions (33). The cross-cutting UMNs identified in Q2 are based on recent feedback and opinions from public health stakeholders gathered by the European Federation of Pharmaceutical Industries and Associations (EFPIA) during recent consultations (27, 34). Other survey questions have been developed as a follow-up to previous monitoring activities conducted within a EC Joint Research Centre (JRC) project, which aimed to explore the scientific and societal impact of biomedical research funded in the EU (35, 36).

Under the ‘General Details’ section, for some questions (‘What is your area of work?’ and ‘What is your primary role?’), and the ‘Survey’ section, for Q1 (‘*In your opinion, what are the most urgent public health challenges today?*’), Q2 (‘*In your opinion, what are the most relevant unmet (bio)medical needs that deserve prioritisation in the research and policy agenda (at member states and EU level)?*’), Q4 (‘*What specific research activities do you think deserve more investment/resources to better face the aforementioned public health challenges and unmet medical needs?*’), and Q5 (‘*Concerning research design, in your opinion what are the approaches/tools that could be more effective to gain research translational success and ultimately public health impact?*’), participants were allowed to select more than one option, which explains why in some graphs the combined percentages exceed 100%.

For Q3 (‘*To date, the prevalence and incidence of many of the aforementioned diseases remain high. Several factors may contribute to this problem. Can you rate their relevance?*’), Q6 (‘*Concerning drug attrition, several factors may contribute to failures in drug development. Can you rate their relevance?*’), and Q7 (‘*Some policy interventions at EU or member state level could be envisaged to tackle public health emerging/unsolved challenges or other unmet biomedical needs. Could you rate their effectiveness?*’), participants were asked to rate provided factors (or policy interventions) selecting either ‘Irrelevant,’ ‘Somewhat relevant,’ ‘Relevant,’ ‘Highly relevant’ or ‘Not sure’ (for Q3 and Q6), and either ‘Not effective,’ ‘Somewhat effective,’ ‘Effective,’ ‘Most effective,’ or ‘Not sure’ (for Q7).

Survey contributions were anonymous, except to surveyors in case the participant expressed the willingness to be contacted again for future activities, thus disclosing name and email.

### 2.2 Survey dissemination and audience selection criteria

The link to the survey was first shared by HSI with roundtable invitees (starting from September 25th, 2023), and some of them further disseminated the survey link through official channels of their respective organisations. The survey link was also posted in the EU Health Policy Platform managed by the Directorate-General for Health and Food Safety (DG SANTE) and on social media (LinkedIn, Twitter and Facebook). Additional outreach emails were sent to 103 major European public health and biomedical research associations and institutions. In addition, to increase the number of respondents, an approach for using PubMed to generate large email lists of potential participants was considered, as previously described (37) (see Supplementary material 2). Owing to the diverse outreach approaches employed, it was not feasible to ascertain the exact extent of the target audience, i.e., the potential respondents who encountered the survey link and recruitment message. Consequently, calculating a response rate was not possible. As of January 22nd, 2024, 148 respondents participated in the survey.

### 2.3 Data analysis and presentation

Survey results were organised, analysed and plotted using the EU Survey Platform analytical tool and Microsoft Office Excel.

### 2.4 Online roundtable

The online roundtable convened by HSI on November 24th 2023 was hosted on the Microsoft Teams platform and initial findings from the survey were shared. Attendees were subsequently divided into three virtual breakout rooms where they were invited to provide feedback on a set of questions (see Supplementary material 2). To increase openness, the discussion was conducted in accordance with the *Chatham House Rule:* anyone who participates in a meeting is free to use information from the discussion, but is not allowed to reveal who made any particular comment. The online roundtable discussion was recorded and transcribed to draft the minutes shared with roundtable participants and the present report.

## 3 Part I: survey findings

### 3.1 Information about survey participants

**Figure 1:**
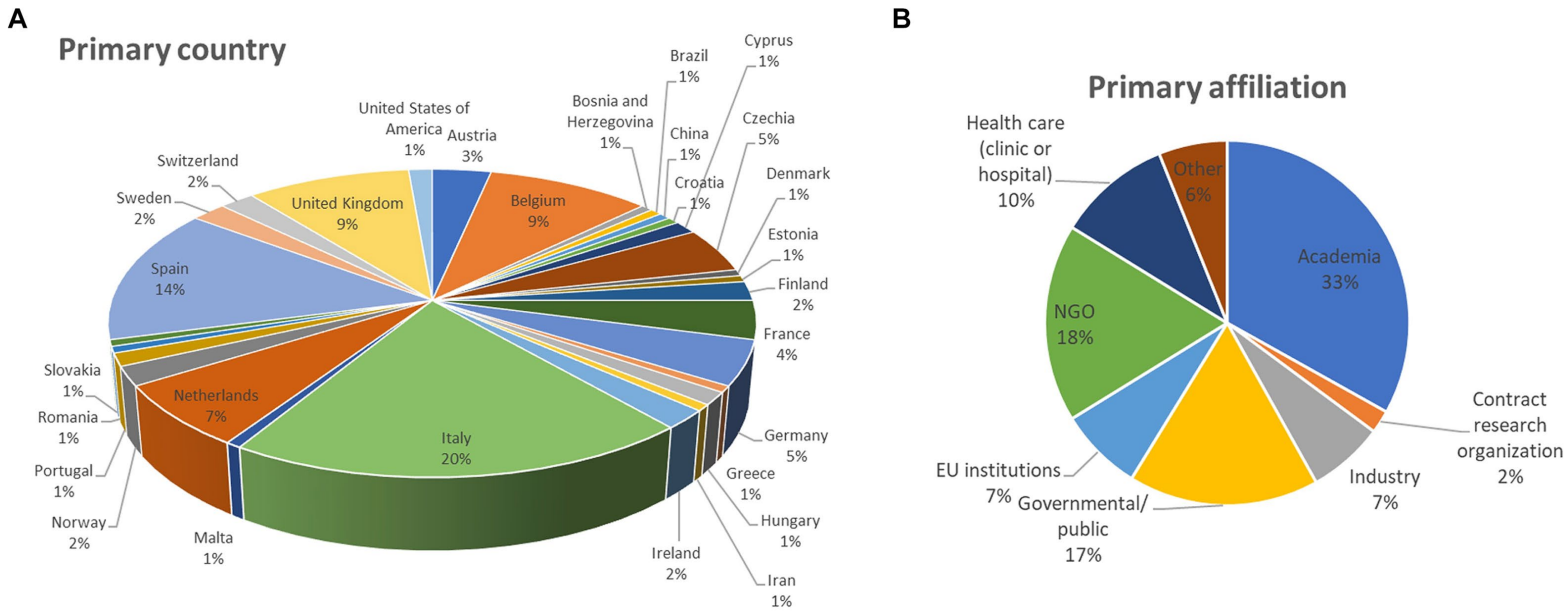
Details about survey participants: **(A)** primary country of work and **(B)** primary affiliation category (percentage of respondents are reported).

Out of the 148 participants who replied to the survey (from September 25th 2023, to January 22nd 2024), about 20% are from Italy, 14% from Spain, an equal 9% from Belgium and the United Kingdom (UK), 7% from the Netherlands, an equal 5% from Germany and Czechia, 4% from France, and 3% from Austria, among other countries (Figure 1A). With the exception of the UK, very few representatives of non-EU countries participated in the survey (each non-EU country was represented on average by about 1% of participants).

When looking at their primary affiliation, different stakeholder categories were heterogeneously represented: 33% are from academia, NGO representatives are 18, 17% indicated governmental and public organisations, 10% of respondents indicated healthcare (clinic or hospital), 7% selected EU institutions, 7% from industry, 2% are from contract research organisations, while 6% indicated “other” (Figure 1B).

**Figure 2:**
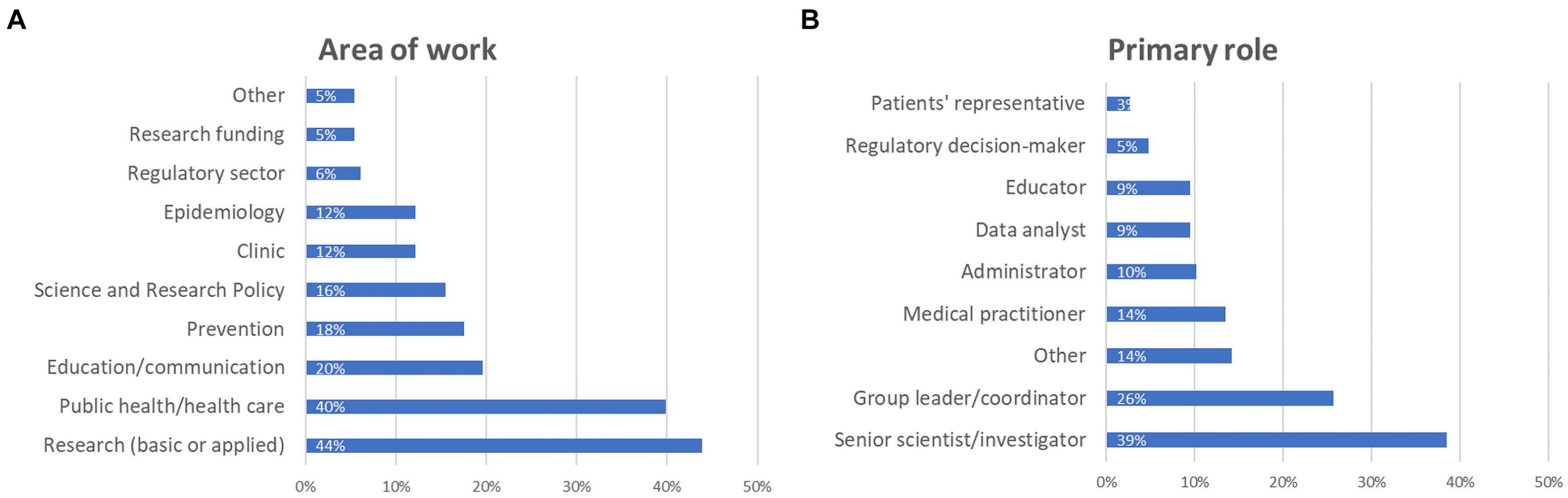
Details about survey participants: **(A)** main area of work, and **(B)** primary role (percentage of respondents are reported). Participants were asked to select at least one option, which explains why the combined percentages exceed 100%.

Regarding participants’ primary area of work, 44% declared that they work in basic or applied research, nearly 40% selected public health and/or health care, almost 20% indicated education/communication, 18% prevention, 16% science and research policy, an equal 12% work on epidemiology and clinic, representatives of the regulatory sector and funding bodies represented 6 and 5%, respectively, while 5% fell outside these categories (Figure 2A).

When considering their primary role, almost 39% are senior scientists/investigators, about 26% group leaders/coordinators, 14% medical practitioners, 10% administrators, an equal 9% are data analysts and educators, about 5% are regulatory decision makers, while almost 3% are patient representatives (Figure 2B). Among the 14% who replied “other” (21 participants in total), a couple indicated CEO, someone indicated environmental health, occupational health or food safety, and other roles were lab technician, nurse, auditor, inspector, external affairs, customer care, and PhD student.

### 3.2 Public health challenges and unmet medical needs

**Figure 3:**
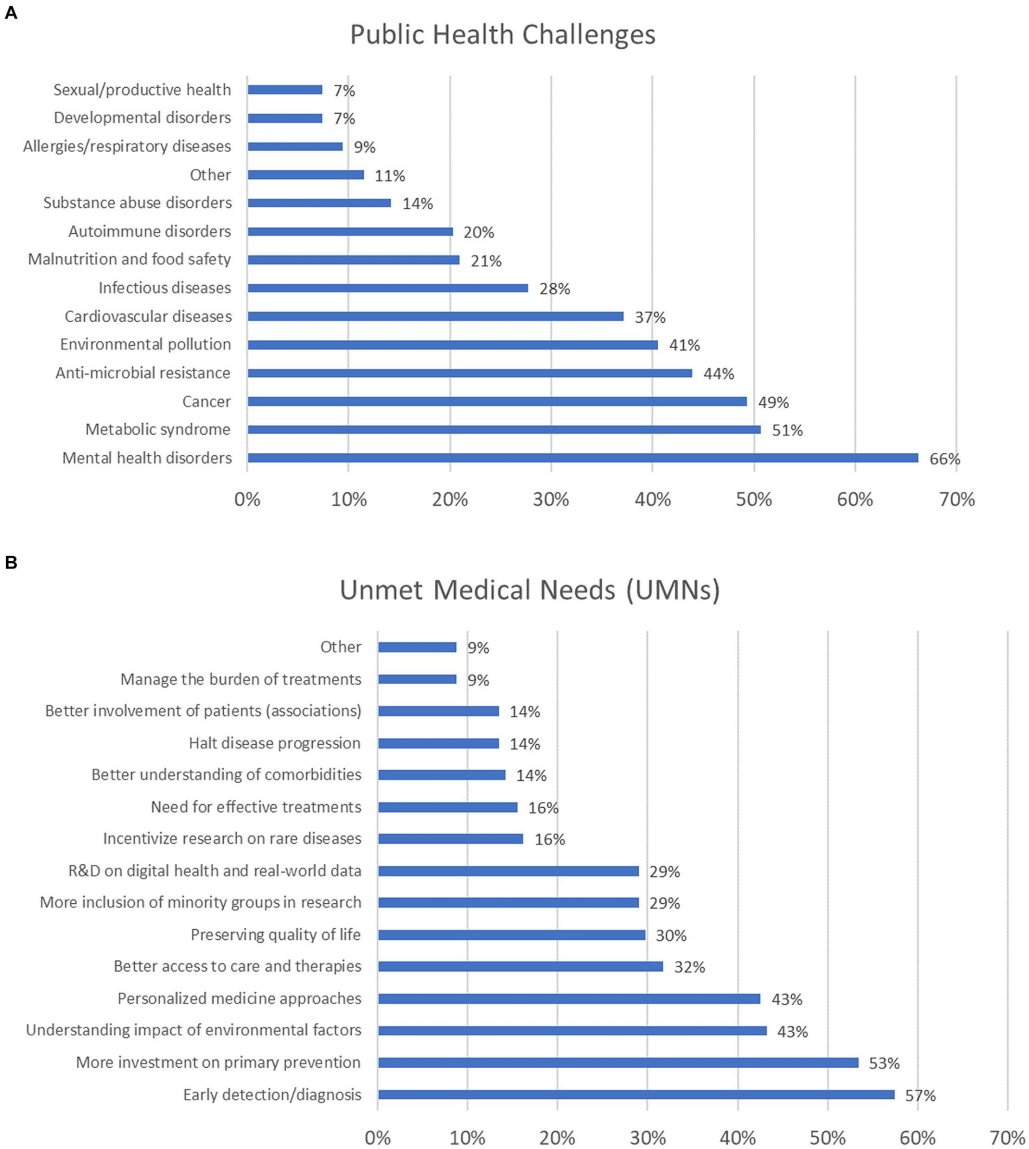
Public health challenges **(A)** and unmet medical needs (UMNs) **(B)** deserving prioritisation (percentage of respondents are reported). For these questions, participants were allowed to select between 1 and 5 options, which explains why the combined percentages exceed 100%.

The survey’s first question aimed to explore which public health challenges deserve prioritisation. Mental health disorders (including depression, anxiety, schizophrenia, and dementia) were selected by more than 66% of participants. Nearly 51% selected metabolic syndrome disorders, 49% opted for cancer, 44% selected antimicrobial resistance (AMR), and 41% opted for environmental pollution. About 37% considered cardiovascular diseases a priority, and 28% did so for infectious diseases. About 21% opted for malnutrition and food safety, 20% considered autoimmune disorders, 14% selected disorders associated with substances of abuse, and 9% considered allergies and respiratory diseases. An equal 7% opted for developmental and sexual/reproductive disorders (Figure 3A). Other public health topics were also indicated, as reported in Supplementary material 2.

When examining the distribution of responses across the six major stakeholder groups (i.e., academia, NGOs, government/public organisations, healthcare, EU institutions, and industry), mental health disorders were most frequently selected by representatives from academia, NGOs, government/public organisations, and EU institutions. In contrast, cancer was the top choice for representatives from both healthcare and industry (the healthcare sector representatives equally prioritised cardiovascular diseases) (Supplementary Figure 1). The second question was about the most relevant UMNs that should be considered nowadays. More than 57% considered that early detection and diagnosis (e.g., aimed at identifying early biomarkers of diseases, screening and secondary prevention) deserve more funding; about 53% of participants selected primary prevention; an equal 43% opted for better understanding the impact of environmental factors in the onset of diseases, and personalised medicine approaches. Better access to care and therapies was chosen by 32% of respondents; 30% considered preservation of life quality for those already affected by diseases; an equal 29% opted for better inclusion of ethnic minority groups (considering, e.g., different socio-economic background, education and genders) in research, and research and development on digital health and real-world data. In addition, an equal 16% considered rare diseases and the lack of effective treatments, while an equal 14% selected (i) better understanding comorbidities, (ii) halting progression of diseases, and (iii) better engagement with patients’ associations (Figure 3B). Other UMNs were suggested, as reported in Supplementary material 2.

Analysis of UMN selection across the six main stakeholder groups showed that increased investment in early detection and diagnosis was the top choice for representatives from academia and EU institutions, primary prevention research was most favoured by representatives from NGOs and government/public organisations (the latter also equally prioritised ‘better understanding the impact of environmental factors’), while personalised medicine was the preferred option among healthcare and industry representatives (Supplementary Figure 2).

### 3.3 Factors responsible for disease prevalence and incidence

The third question enquired about what factors could mainly contribute to prevalence and incidence of diseases. Participants were asked to rank the enlisted factors, assigning a relevance score (from “highly relevant” to “irrelevant,” with the possibility to select “not sure” in case they had no clear opinion).

When considering the five most selected factors that were rated as “highly relevant,” 55% of respondents opted for the insufficient investment in primary prevention; 51% considered the inefficient implementation of prevention strategies; low public awareness about risk factors was considered by 41% of respondents; 39% considered the insufficient (or lack of) funding in some research areas; and 33% opted for lack of investment in disease aetiology and epidemiology.

**Figure 4:**
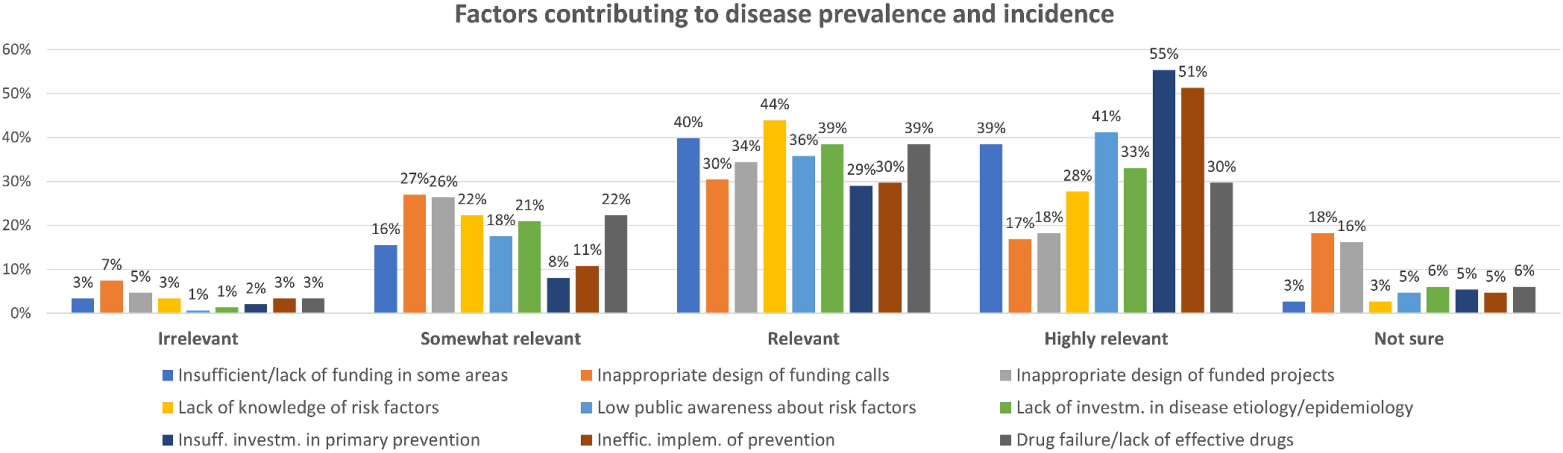
Factors contributing to disease prevalence and incidence: participants could rate each factor either as ‘Irrelevant,’ ‘Somewhat relevant,’ ‘Relevant,’ ‘Highly relevant’ or ‘Not sure’ (percentage of respondents are reported).

When looking at the five most selected factors considered as “relevant” in the contribution of disease prevalence and incidence, 44% believe that the lack of knowledge about novel or emerging contributing risk factors deserves attention; almost 40% selected the insufficient or lack of investment and funding in some specific disease areas; an equal 39% considered the lack of investment in epidemiology and disease aetiology, and the lack of effective drugs (and drug failure); and 36% chose low public awareness about risk factors (Figure 4). Other factors were also indicated, as reported in Supplementary material 2.

### 3.4 Initiatives warranting increased funding to address public health challenges and UMNs

**Figure 5:**
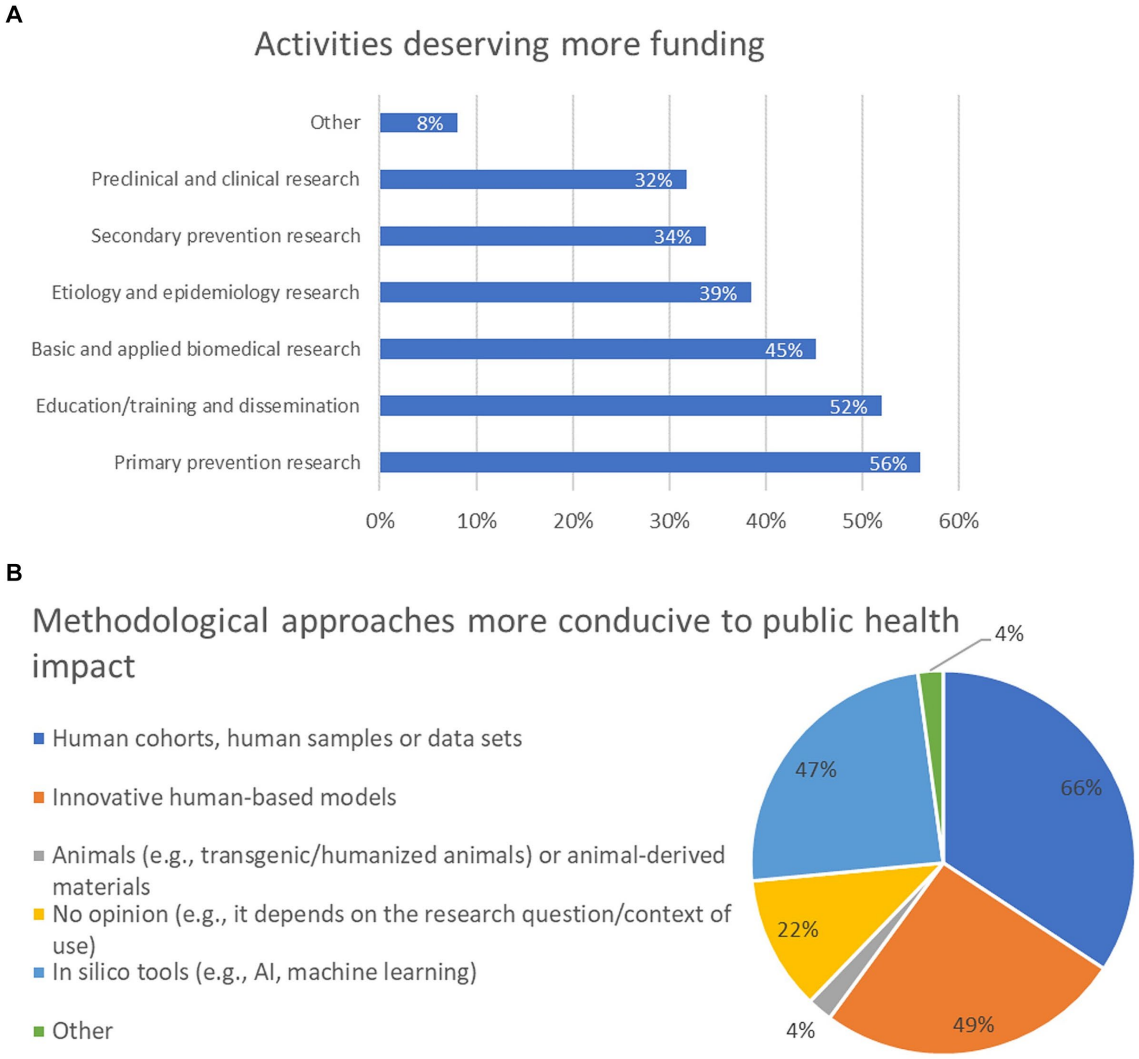
Activities deserving more funding to face public health challenges and unmet medical needs (UMNs) **(A)**, and methodological approaches in research activities that could be more conducive to public health (societal) impact **(B)** (percentage of respondents are reported). For panel **(A)**, participants were allowed to select between 1 and 3 options, whilst for panel **(B)**, participants had no limit with the selection of the options. For this reason, the combined percentages in these graphs exceed 100%.

The fourth question investigated what activities would deserve more funding to face public health challenges and UMNs. About 56% of participants think that primary prevention research (e.g., through observational or intervention studies) deserves better funding; 52% opted for education/training and dissemination (e.g., education or public outreach activities); 45% selected basic and applied biomedical research (e.g., to investigate novel disease mechanisms and identify new druggable targets); 39% considered aetiology and epidemiology research (e.g., to investigate still unknown risk factors); 34% prioritised secondary prevention research (e.g., to design or implement the use of novel diagnostic and screening tools/devices). In addition, preclinical and clinical research was selected by 32% of participants (Figure 5A). Other topics were also proposed by 8% of participants, as reported in Supplementary material 2.

Analysis of replies across the six major stakeholder categories showed that increased funding on primary prevention research was the most selected option by stakeholders from academia and government/public institutions; representatives from NGOs prioritised education/training and dissemination activities; basic and applied research was the most selected option by representatives from the healthcare sector; EU institutions’ representatives mostly preferred secondary prevention research, and research on etiology and epidemiology was the most selected option by the industry representatives (Supplementary Figure 3).

### 3.5 Research approaches with potential for societal impact and factors contributing to drug failure

The fifth question investigated what research methodological approaches could be more conducive to societal impact. Nearly 66% of respondents think that human cohorts, the use of human samples and/or data sets, in general, should be prioritised; 49% opted for innovative human-based *in vitro* models (e.g., complex cellular/tissue/organ models); 47% selected *in silico* tools, AI and machine learning, while 22% have no opinion and think that the selection of the method depends on the research question and the context of use. Four percent of respondents believe that animals (e.g., transgenic/humanised animals) or animal-derived materials should be considered (Figure 5B). Analysis across the six major stakeholder categories revealed that human cohorts, human samples and/or data sets was the most selected option for all stakeholder groups, with the exception of representatives from industry, who prioritised *in silico* tools (Supplementary Figure 4).

**Figure 6:**
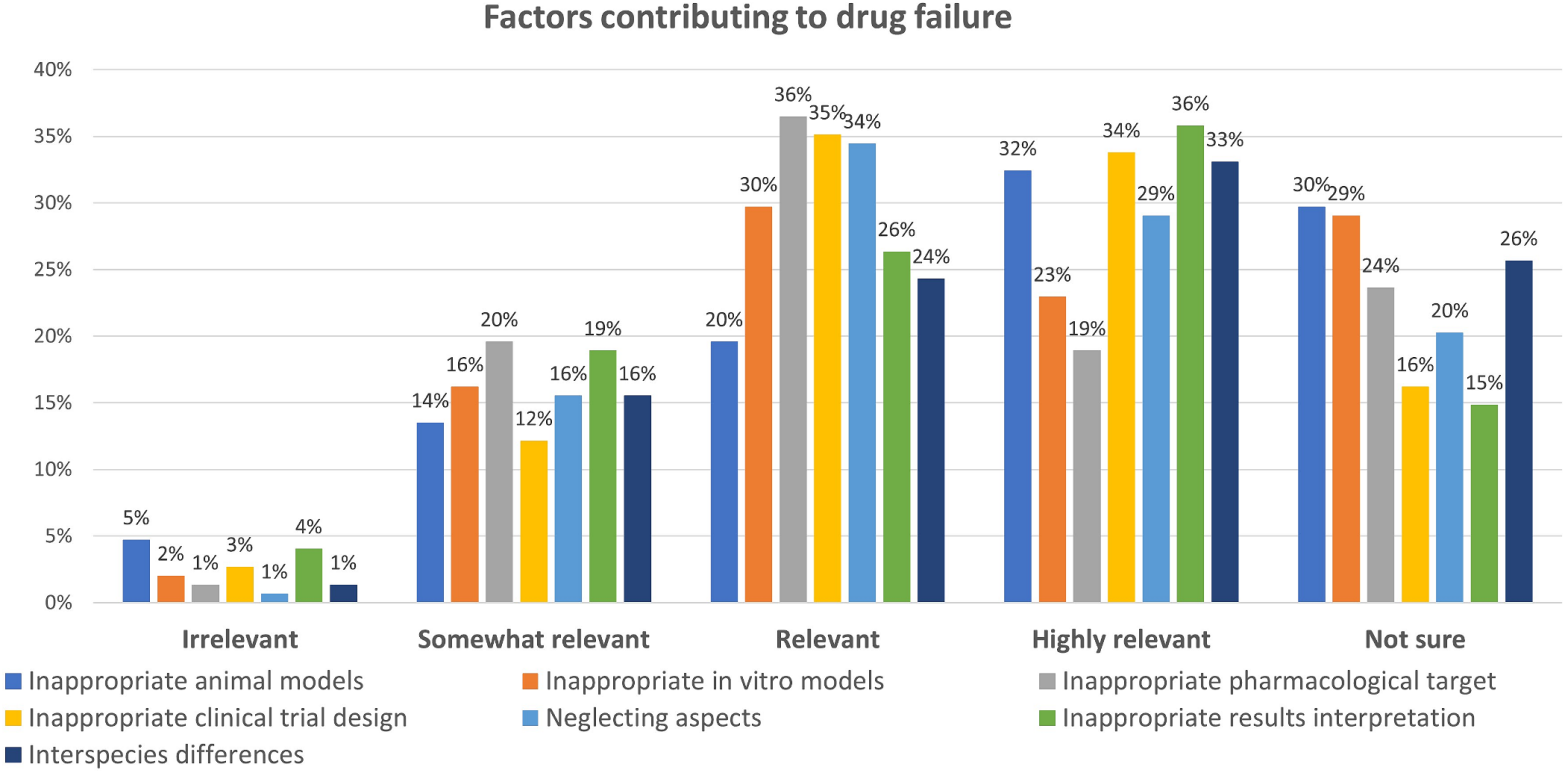
Factors contributing to drug failure: participants could rate each factor either as ‘Irrelevant,’ ‘Somewhat relevant,’ ‘Relevant,’ ‘Highly relevant’ or ‘Not sure’ (percentage of respondents are reported).

Question six enquired about the factors mainly contributing to drug failure. Also in this case, participants were asked to rank the options provided according to their relevance, from “highly relevant” to “irrelevant,” and with the possibility to opt for “not sure” (Figure 6).

When looking at the five most selected factors considered as “highly relevant,” 36% opted for the inappropriate interpretation of research results, 34% for the inappropriate clinical trial design (e.g., biased selection or low number of participants), 33% considered the inter-species differences responsible for lack of efficacy or toxicity, 32% selected the inappropriate choice of *in vivo* (animal) models at research or preclinical stage, and 29% believe that neglecting pharmacokinetics or pharmacodynamics when designing clinical trial or when interpreting results plays a role.

Regarding the five most selected factors considered as “relevant” contributors to drug failure, 36% opted for the wrong or inappropriate target selection, 35% chose the inappropriate clinical trial design, 34% think that neglecting pharmacokinetics or pharmacodynamics aspects plays a role, 30% considered the improper selection of *in vitro* (animal- or human-derived) models at research or preclinical stage, while wrong interpretation of research results was rated as “relevant” by 26% of respondents.

Notably, 29–30% of respondents express uncertainty regarding the potential impact of inappropriate *in vitro* or *in vivo* models on drug failure, while 26% are unsure about the significance of interspecies differences in this context (Figure 6). In addition, other comments were provided (see Supplementary material 2).

### 3.6 Suitable policy intervention strategies to address public health challenges and UMNs

**Figure 7:**
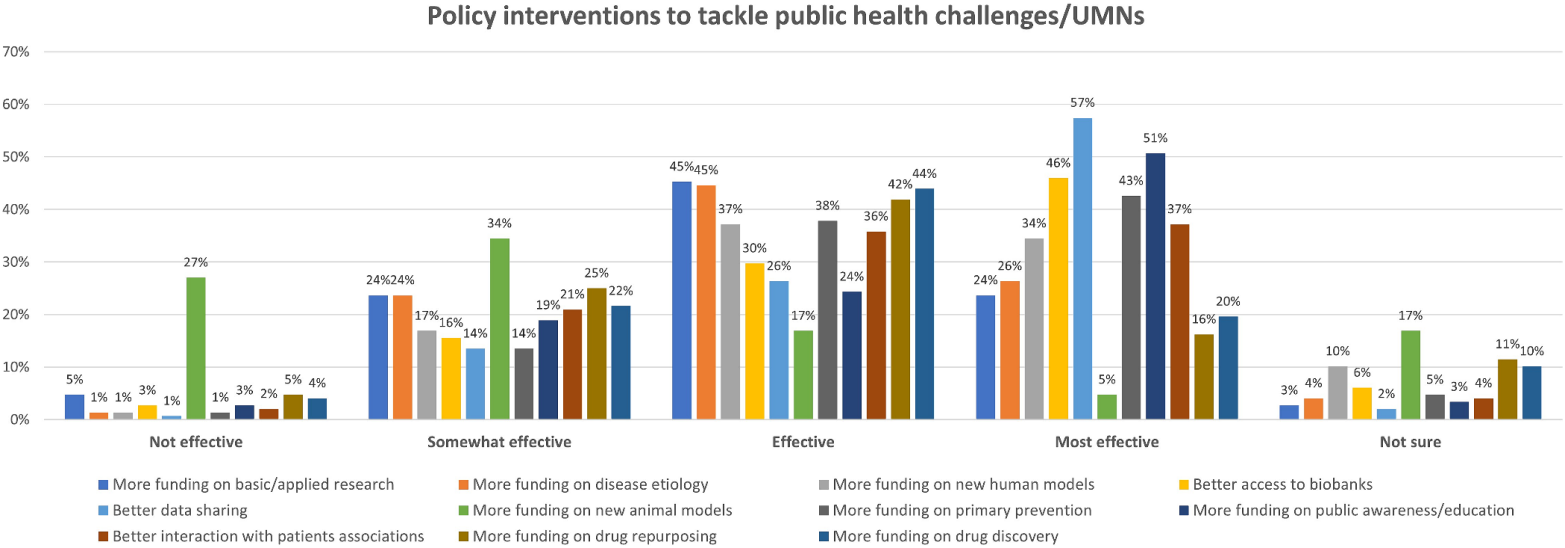
Possible policy intervention strategies to tackle public health emerging/unsolved challenges or other UMNs: participants could rate each policy intervention either as ‘Not effective,’ ‘Somewhat effective,’ ‘Effective,’ ‘Most effective,’ or ‘Not sure’ (percentage of respondents are reported).

The last question of the survey aimed to explore what policy intervention strategies could be envisaged to tackle emerging or unsolved public health challenges or other UMNs. As for previous questions, respondents were asked to rate their effectiveness from “most effective” to “not effective” or to choose “not sure” in case of uncertainty (Figure 7). When considering the five most selected interventions rated as “most effective,” we found that 57% think that better data sharing should be prioritised; 51% opted for the need to allocate more funding to public awareness, education, dissemination activities; 46% think that improving research institutes’ access to biobank should be considered; 43% believe that increasing funding on primary prevention research (e.g., observational and intervention studies in human cohorts) should be prioritised; while 37% believe that better dialogue with patients’ associations about UMNs and research priorities should be supported.

These are the five most selected research policy interventions ranked as “effective”: an equal 45% opted for the need to increase funding for basic and applied research (e.g., to identify novel disease mechanisms) and for the identification of novel etiological factors (e.g., environmental or genetic risk factors); 44% opted for increased funding on new drug discovery; 42% selected drug repurposing; and 38% considered primary prevention research.

Notably, 27% of participants considered that allocating more funding to biomedical projects focused on innovative animal models (e.g., humanised mouse models) can be considered an “ineffective” course of action, while 34% believe that this could be “somewhat effective” (Figure 7). Other policy options were also suggested by some participants (Supplementary material 2).

At the end of the survey, 61% of participants expressed their willingness to continue contributing to this scoping activity.

### 3.7 Possible research-policy interventions based on survey’s findings

Based on survey results, the following 11 research-policy interventions (RPI) could be considered to address the public health challenges and UMNs that have been prioritised by survey participants:

- RPI 1: Increase investment in **primary prevention**;
- RPI 2: Increase implementation of **prevention strategies**, including education/training and dissemination;
- RPI 3: Foster **public awareness** about risk factors, allocating more funding to education, dissemination activities;
- RPI 4: Increase funding in research on **novel disease mechanisms** and new druggable targets, disease **etiology** and **epidemiology**;
- RPI 5: Prioritise **innovative human-relevant** *in vitro* and ***in silico* approaches** in research;
- RPI 6: Allocate more resources on **secondary prevention** research (e.g., to design/implement novel diagnostic and screening tools/devices);
- RPI 7: Allocate more resources on human-relevant **preclinical research** as well as **clinical research**;
- RPI 8: Improve **clinical trial design**, accounting for more heterogenous cohorts and considering PK/PD aspects;
- RPI 9: Increase **data sharing**;
- RPI 10: Improve **access to biobanks**;
- RPI 11: Incentivise **better dialogue** with patients’ associations to define priorities.

### 3.8 Survey’s limitations

Conducting research surveys necessitates careful consideration due to the inherently subjective nature of the information collected. In the case of this survey, feedback was garnered from a relatively modest cohort of participants (148 respondents), which complicates the task of deriving quantitative insights regarding the subjects under investigation. The inability to calculate a response rate due to diverse outreach approaches represents another limitation of this survey. Considering the number of emails sent by HSI (about 2,940), the further dissemination of survey link by roundtable invitees, the posting of the survey link on the EU Health Policy Platform and social media channels, and the final number of respondents, it can be estimated that the response rate was low. An additional drawback is the impossibility to assess the associated potential nonresponse bias (i.e., nonresponse to the survey by potential participants in a target group) (38). While considering that participation rates have generally declined across all survey modes over the past decade (39) and being mindful of identified limitations, survey results have illuminated some critical public health topics, UMNs, and possible RPIs deserving attention.

## 4 Part II: multistakeholder roundtable discussion

At EU level, several research and policy initiatives have been launched in recent years that are in line with the aforementioned RPIs. Recent funding programmes, especially Horizon 2020 (H2020) and Horizon Europe, have included important calls for health research proposals aimed at enhancing the impact of biomedical research findings, enabling faster exploitation of research results and their translation into health policies. These aspects were discussed in an online roundtable hosted by HSI with representatives across various stakeholder groups, and are reported in the next sections.

### 4.1 Public health challenges and UMNs warranting prioritisation (linked to RPIs 1, 2, 4, 5, 7, 8)

Survey replies showed that mental health disorders, metabolic syndrome, cancer, AMR, environmental pollution, and cardiovascular diseases are the most relevant public health challenges deserving prioritisation. Cancer, metabolic syndromes, diabetes, cardiovascular diseases and mental health disorders are indeed highly prevalent worldwide, are often associated with other co-morbidities (7–10, 40, 41), and represent a heavy burden also for the healthcare systems (11, 42).

Noteworthy, AMR has been identified as one of the top three EU health threats (43), straining healthcare systems and society (44).

In addition, some stakeholders who participated in the roundtable suggested that neurodegenerative diseases deserve special attention in light of their high complexity and heterogeneity (45) and the lack of effective treatments for many of these diseases, including Alzheimer’s disease (46).

Others considered that also sexual and reproductive health warrants prioritisation, in light of its broad impact and implications (47, 48).

Among the UMNs considered by survey participants, early detection and diagnosis, primary prevention, a better understanding of the impact of environmental factors in the onset of diseases, and personalised medicine approaches were the most selected topics (in this order). Other popular options include better access to care and therapies, preservation of life quality for those already affected by diseases, better inclusion of ethnic minority groups in research, and research and development on digital health and real-world data.

Recent EU policy initiatives and research funding strategies have addressed some of the aforementioned public health topics and UMNs (see Table 1). However, more research efforts should be made to investigate the role of primary prevention to reduce the burden of cardiovascular diseases and the associated metabolic syndrome comorbidities, including obesity (49).

**Table 1:** EU initiatives, programs and funding calls that have been launched in recent years and that are directly or indirectly linked to the research-policy interventions (RPIs) derived from survey findings, and some actionable recommendations aimed at facilitating research-into-policy translation.

Topics	EU initiatives, programs and funding calls	Survey-derived RPIs that are directly/indirectly linked to these initiatives	Possible actionable recommendations to facilitate research-into-policy translation
Public health challenges and UMNs warranting prioritisation	Cancer EU Mission is supported within the Health cluster.	RPI 1: Increase investment in **primary prevention.** RPI 2: Increase implementation of **prevention strategies**, including education/training and dissemination.	R1: Prioritise research on primary prevention strategies to reduce the burden of NCDs and associated comorbidities R2: Dedicated calls for proposals should be initiated to investigate the impact of environmental pollution on disease onset.
	Several EU funding calls have addressed cardiovascular disease research, such as EU research on cardiovascular diseases calls and ERA-CVD.		
	Mental health has been considered a top priority by DG SANTE; recent flagship initiatives addressing prevention and early intervention for mental health problems have been launched (COM(2023) 298 final).		
	A recent One Health Conference (organised by DG SANTE in November 2023) emphasised the need to tackle AMR.		
	AMR has been addressed in the Reform of the EU pharmaceutical legislation.		
	Cancer and AMR have been the focus of several EIC-funded health projects (EIC 2023 work programme).		
	EU initiatives have focused on neglected infectious diseases, such as EDCTP.	RPI 2: Increase implementation of **prevention strategies**, including education/training and dissemination.	R3: Encourage participative collaboration with low-middle income countries to effectively address emerging public health threats and environmental concerns
	Europe’s beating cancer plan and European Partnership for Personalised Medicine addressed personalised medicine.	RPI 4: Increase funding in research on **novel disease mechanisms** and new druggable targets RPI 5: Prioritise **innovative human-relevant** *in vitro* **and *in silico* approaches** in research RPI 7: Allocate more resources on human-relevant **preclinical research** as well as **clinical research** RPI 8: Improve **clinical trial design**	R4: Prioritise initiatives addressing the lack of medicines for rare diseases, children, and pregnant women.
Prevention and early diagnosis	Ageing-related issues have been investigated at EU level in recent years, e.g., in JPND Neurodegenerative Disease Research, the NU-AGE project, ERC Proof of Concept Grants for UNBIAS, a call on Personalised blueprint of chronic inflammation in health-to-disease transition.	RPI 4: Increase funding in research on **novel disease mechanisms** and new druggable targets, disease **etiology** and **epidemiology** RPI 6: Allocate more resources on **secondary prevention** research (e.g., to design/implement novel diagnostic and screening tools/devices)	R5: Efforts to improve early detection of disease among women should be put in place.	
	The OECD has elaborated a conceptual framework to address the Economics of Prevention.	RPI 1: Increase investment in **primary prevention.** RPI 2: Increase implementation of **prevention strategies**, including education/training and dissemination.	R6: Develop additional tools and mechanisms to demonstrate the economic benefits of prevention. R7: MS should implement prevention strategies based on the latest scientific evidence and ensure accountability. R8: Allocate more funding on personalised prevention strategies to prevent the onset, progression, and recurrence of diseases	
	The recently funded PROPHET project focuses on personalised prevention.				
	A recent Horizon RIA call focuses on Personalised prevention of NCDs, addressing areas of unmet needs using multiple data sources HORIZON-HLTH-2024-STAYHLTH-01-05-two-stage.		
	Two EP resolutions focus on NCDs and Mental health and advocate for implementing prevention research and measures to alleviate the burden of these conditions.		
	Recent Marketplaces events organised by the DG SANTE focused on prevention and patient-centric approaches.		
Public awareness, education and training of healthcare actors	Schools4Health led by EuroHealthNet and funded under the EU4Health programme, seeks to promote, enhance, and maintain the implementation of a collaborative, school-wide strategy for health and wellbeing.	RPI 2: Increase implementation of **prevention strategies**, including education/training and dissemination RPI 3: Foster **public awareness** about risk factors, allocating more funding to education, dissemination activities	R9: Include comprehensive nutrition education in medical school curricula to ensure that future physicians are equipped with the knowledge and skills to provide practical nutritional advice. R10: Improve individual protective behaviour and address systemic drivers of harmful behaviour to tackle societal challenges
	Medical practitioners’ education has been discussed at a recent Webinar organised by the European Forum for Primary Care (EFPC).		R11: Reform medical practitioners’ education to include training that addresses multi-morbidities and emphasises person-centred care.
	Recent EC calls emphasise the importance of social sciences and humanities and citizen/patient driven co-creation efforts, e.g., HORIZON-HLTH-2024-TOOL-05-06-two-stage, HORIZON-HLTH-2024-TOOL-11-02, and HORIZON-JU-IHI-2023-05-01.	RPI 11: Incentivise **better dialogue** with patients’ associations to define priorities.	R12: Involve educators, patients and patient associations to integrate their view into primary healthcare.
Impact of environmental pollution on health	The European Green Deal and the Roadmap for moving to a competitive low carbon economy in 2050 have the potential to inform evidence-based policies and funding to effectively address interconnected threats such as pollution, climate change, and health.	RPI 2: Increase implementation of **prevention strategies**, including education/training and dissemination. RPI 3: Foster **public awareness** about risk factors, allocating more funding to education, dissemination activities RPI 4: Increase funding in research on **novel disease mechanisms** and new druggable targets, disease **etiology** and **epidemiology**	R13: Align international policies and interventions to reduce the burden of pollution on humans and animals, and ensure clean and healthy environments for all. R14: Invest in the development of tools and methods to better understand and address the impact of chemicals, viral infections, and other environmental factors on human health.
	European Partnerships in health under Horizon Europe and the Health Cluster address several gaps and challenges for health and care, including environment and health.		
	A high-level conference organised by DG RTD addressed climate change impact on health.		
Clinical trial design	The IHI call 4 topic focuses on Inclusive clinical studies for equitable access to clinical research in Europe.	RPI 8: Improve **clinical trial design**	R15: Increase support for clinical studies addressing equitable access and inclusivity and leveraging AI technologies. R16: Develop training courses for clinicians and scientists on proper clinical research design.
	IHI programmes have taken steps to improve the design of clinical trials and data quality through a portfolio of projects, such as EU-PEARL, Connect 4 children, and Trials@home.		R17: Support research on vulnerable populations to understand gender differences in treatment responses and identify (epi)genetic disease susceptibilities.
Data sharing and data quality	Some IHI projects aim to facilitate data sharing and curation, e.g., BigData@Heart; DO-IT; EHDEN, and the reproducibility of research data, e.g., EQIPD.	RPI 9: Increase **data sharing**	R18: Increase efforts to promote the curation, standardisation, and harmonisation of health data. R19: Provide support for the datafication process of individual biobanks to enhance accessibility to samples and data across EU.
	Rare Disease Monitor is a pilot project that sees the collaboration between NFU and the Elsevier group to offer public access to information (incl. articles, metadata, and other insights, regarding rare (or orphan) disease research activities in the Netherlands).		
	BBMRI-ERIC supports the establishment of European Open Science Cloud – EOSC.	RPI 9: Increase **data sharing** RPI 10: Improve **access to biobanks**	
	DG RTD has launched several calls on data-driven approaches to optimise patient outcomes in several chronic diseases, such as the H2020 call on Use of Real-World Data to advance research on the management of complex chronic conditions.	RPI 5: Prioritise **innovative** human-relevant *in vitro* **and *in silico* approaches** RPI 9: Increase **data sharing**	
	Applying the FAIR data principle (findability, accessibility, interoperability, and reusability) is a requirement, especially in data-intensive projects.	RPI 9: Increase **data sharing**	
	The H2020 Open Access manual describes standardisations and optimisation of semantic and ontological metadata models for AI or machine-readable approaches.	RPI 5: Prioritise **innovative** human-relevant ***in vitro* and *in silico* approaches** in research RPI 9: Increase **data sharing**	
	Reuse of data has been emphasised under Action 2 of the European Research Area (ERA) Policy Agenda.	RPI 9: Increase **data sharing**	R20: Allocate a significant portion of new funding to projects that emphasise data reusability and build on previously released data.
	BBMRI-ERIC participates in EHDS2 Pilot project promoting the **secondary use** of health data.	RPI 9: Increase **data sharing** RPI 10: Improve **access to biobanks**		
	The most recent EU Digital Act aims to create a safer digital environment that protects users’ fundamental rights and ensures a level playing field for businesses.	RPI 5: Prioritise **innovative** human-relevant ***in vitro* and *in silico* approaches** in research RPI 9: Increase **data sharing**	R21: Reduce data access restrictions in the health sector for AI applications and data sharing. R22: Enhance curation efforts for integrating personal and patient clinical data.
	The European Health Data Space regulation empowers individuals to manage their health data, promotes the utilisation of health data for improved healthcare delivery, research, innovation and policymaking, and facilitates the EU in harnessing the full potential of secure and safe exchange, use, and reuse of health data.	RPI 9: Increase **data sharing**	
	EMA and HMA have set up the Big Data steering group, which aims to make sense of big data and real-world data and how to use them to improve regulatory approaches.	RPI 5: Prioritise **innovative** human-relevant ***in vitro* and *in silico* approaches** in research RPI 9: Increase **data sharing**	
	The Data Analysis and Real World Interrogation Network (DARWIN EU) delivers real-world evidence from across the EU on diseases, populations, and the uses and performance of medicines, helping regulators fill data gaps.	RPI 5: Prioritise **innovative** human-relevant ***in vitro* and *in silico* approaches** in research RPI 9: Increase **data sharing**	
	The Accelerating Clinical Trials in the EU (ACT EU) discusses how to optimise multinational clinical trials.	RPI 8: Improve **clinical trial design** RPI 9: Increase **data sharing**	
	Some alternative publishing models, such as F1000Research and Journal of Negative Results in BioMedicine allow preprint and publication of so-called ‘negative results’	RPI 9: Increase **data sharing**	R23: Editors should rigorously select reviewers for peer-reviewed journals to ensure critical assessment of manuscripts. R24: Incentivise publication and data sharing of findings that contradict original research hypotheses or previous evidence.
Supporting innovation in biomedical research	Over the last 20 years, the EU has allocated over 1B euros to >300 projects to develop new approach methodologies; the industrial sector has also supplemented this endeavour by contributing an extra 150 M euros (ECI Save Cruelty-Free Cosmetics).	RPI 5: Prioritise **innovative human-relevant *in vitro* and *in silico* approaches** in research RPI 7: Allocate more resources on human-relevant **preclinical research** as well as **clinical research** RPI 8: Improve **clinical trial design**	R25: Allocate funds to validate and benchmark innovative models and methods to increase confidence in their use and support implementation science. R26: Enhance accessibility to biobanks and data repositories, allowing for individualised setups, to facilitate the adoption of personalised medicine approaches. R27: Further assess the clinical utility of new approaches with dedicated funding. R28: Streamline the regulatory approval process for AI technologies by involving regulators earlier in the technology development process	
	Recent research actions call for the submission of proposals focused on: *Innovative non-animal tools and strategies for biomedical research* HORIZON-HLTH-2024-TOOL-05-06-two-stage; *Bio-printing of living cells for regenerative medicine* HORIZON-HLTH-2024-TOOL-11-02; and *implementation of non-animal approaches for the development, testing and production of health technologies* HORIZON-JU-IHI-2023-05-01.				
	A recent IHI funding call topic (Call 7, topic 3) aims to support the clinical validation of both candidate biomarkers and any innovative technologies needed for their use in the clinic (HORIZON-JU-IHI-2024-07-03-singe-stage).		
	The EC Better Regulation Agenda ensures EU policymaking is based on evidence and supports the needs of the regulators.		
	A recent Horizon Europe call aims at gaining experience and confidence in NAMs for regulatory safety and efficacy testing, with coordinated training and experience exchange for regulators (HORIZON-HLTH-2024-IND-06-09).		
	The In Silico World project funded under Horizon 2020 is a prime example of success in streamlining regulatory processes for innovative technologies.		
	Preliminary regulatory acceptance from EMA has been achieved for the UISS-TB-DR simulation platform, a significant milestone for tuberculosis vaccine development.		
Research impact and multidisciplinarity	Recent Horizon Europe calls (e.g., HORIZON-HLTH-2024-STAYHLTH-01-05-two-stage; HORIZON-HLTH-2024-TOOL-11-02; HORIZON-JU-IHI-2023-05-01; HORIZON-JU-IHI-2024-07-03-singe-stage) put strong emphasis on multidisciplinary research, including social sciences and humanities and multi-stakeholder engagement.	RPI 4: Increase funding in research on **novel disease mechanisms** and new druggable targets, disease **etiology** and **epidemiology** RPI 5: Prioritise **innovative human-relevant *in vitro* and *in silico* approaches** in research RPI 6: Allocate more resources on **secondary prevention** research (e.g., to design/implement novel diagnostic and screening tools/devices) RPI 7: Allocate more resources on human-relevant **preclinical research** as well as **clinical research** RPI 8: Improve **clinical trial design**, accounting for more heterogenous cohorts and considering PK/PD aspects RPI 9: Increase **data sharing**	R29: Foster continuing partnerships between academia, industry, policymakers, and regulatory bodies to streamline the application and maximise the impact of research outputs. R30: Establish spaces and platforms to facilitate community bridging in a multi/interdisciplinary manner from project design to implementation.	
	Joint undertakings exemplify fostering interdisciplinarity through public-private partnerships incorporating industrial research in specific sectors.				
	IHI Partners include five European trade associations from the pharmaceutical, medical technology, biotechnology, digital health, and vaccine sectors, supporting multidisciplinary projects.		
	Recent IHI calls focus on: (i) evidence-based practical guidance for sponsors on the use of real-world data/evidence applicable to drugs, medical devices and combinations of both to support decision-making: HORIZON-JU-IHI-2024-06-02-two-stage; (ii) heart disease care, from early detection/diagnosis to treatment through the development of integrated solutions HORIZON-JU-IHI-2024-07-01-single-stage.		
	A recent IHI call focuses on clinical validation of candidate biomarkers as well as any innovative technologies needed for their use in the clinic: HORIZON-JU-IHI-2024-07-03-singe-stage.		R31: Implement further initiatives to support the validation and qualification of newly discovered biomarkers.
	The EIC accelerator program considers market priorities when defining health priorities and the status of innovation in different health areas.		R32: Research projects with clear potential for practical implementation in the healthcare system and market should deserve prioritisation.
	The project Tracking of research results was initiated to identify and reveal the outputs and impacts of funded research under FP7 and H2020 as well as non-FP programmes, 5, 10 and 15 years after implementation, to enable policymakers’ access to a more complete set of information that would contribute to their policy-decision making process.		R33: Robust and reliable indicators should be developed and implemented to monitor the retrospective impact of funded research.
	The EC JRC has conducted recent retrospective analyses of the impact of EU-funded research in some selected disease area: Building indicators to assess the impact of EU-funded research into Alzheimer's disease, breast cancer and prostate cancer		

AI, artificial intelligence; AMR, antimicrobial resistance; DG RTD, EC Directorate General for Research and Innovation; DG, Directorate General; ECI, European Citizen’s Initiative; EFPC, European Forum for Primary Care; EHDS, European Health Data Space; EIC, European Innovation Council; EMA, European Medicines Agency; EP, European Parliament; ERA, European Research Area; ERC, European Research Council; FP, framework programme; H2020, Horizon 2020; HMA, Heads of Medicines Agencies; IHI, Innovative Health Initiative; JPND, Joint Programme – Neurodegenerative Disease Research; JRC, Joint Research Centre; MS, Member States; NAMs, new approach methodologies; NCDs, noncommunicable diseases; OECD, Organisation for Economic Co-operation and Development; RIA, Research Innovation Action; RPI, research-policy intervention.

In addition, several stakeholder representatives highlighted the significant role of environmental pollution in the onset of cardiovascular diseases (50, 51), metabolic syndromes (52), and cancer (53), and believe this area warrants further investigation in research programmes.

Some stakeholders highlighted that also infectious diseases and the impact of climate change deserve attention. For instance, dengue fever is becoming transmissible in Italy and France (54), and although vector-borne diseases may not be a priority at present, they will become an increasing problem in the near future, with habitat and climate changes, as well as other non-climate drivers, such as globalisation, sociodemographics, and changes in public health systems (55, 56). Major EU initiatives have been devoted to neglected infectious diseases (Table 1). In this context, participative collaboration with low-middle income countries that goes beyond ideal project planning is key to face emerging public health threats and tackle the environmental concerns that could lessen climate change.

When considering UMNs, also the lack of medicines for rare diseases, children, and pregnant women should be prioritised, considering that there are very few medicines tested in these populations.

In addition, several survey participants have recognised personalised medicine as a critical UMN, and recent EC initiatives have shifted the focus on this specific topic (see Table 1).

### 4.2 Role of prevention and early diagnosis (linked to RPIs 1, 2, 4, 6)

Survey results suggest that primary prevention research and the inefficient implementation of prevention strategies are primary aspects to address at EU research and policy level.

Almost 80% of the causative factors of NCDs are related to lifestyle, in particular, poor nutrition, smoking and insufficient physical activity (18, 57). An overarching approach, with prevention as a top priority, is highly advisable, considering not just the role played by lifestyle, but also pollution and climate change in preventing, e.g., respiratory diseases, cancers and neurodegenerative diseases (58). In addition, the EU population is progressively ageing, with an increasing number of older people and a decrease in the proportion of working-age people (59), with a predictable increase in healthcare costs.

Ageing-related issues, such as chronic inflammation, have been investigated at EU level in recent years (Table 1). A recent study by the International Longevity Centre (ILC) showed that age, socio-economic status, gender, ethnicity, and geographical location are associated with the likelihood of being diagnosed and receiving treatment for structural heart disease, prompting the need to improve both early detection (especially among women) and therapy (60).

More tools or mechanisms that demonstrate the economic benefits of prevention are needed (as the framework proposed by the OECD, see Table 1); these are essential to put prevention strategies as a forefront public health priority, and influence funding decisions by national ministries along this direction.

Early diagnosis and the identification of biomarkers predictive of disease progression are key, e.g., to identify patients affected by rare diseases (61), or mild cognitive impairment that could possibly lead to dementia (62), enabling timely prevention, reducing healthcare costs, and avoiding frequent hospitalisations and prolonged pharmacological therapies.

It is also essential to reflect on how prevention measures have been or can be implemented, keeping in mind that while research on these topics is supported at the EU level, health practices’ implementation is in the hands of MS. MS governments should actively participate in implementing prevention strategies, and their commitment to this cause and accountability shall be demonstrated. Notwithstanding, preventive medicine and research face insufficient funding within the EU MS, constituting only a minor share of overall health budgets (63).

In addition to citizen education (see section 4.3 for further insights on this aspect), developmental (focusing on personal and social skills) and environmental (focusing on physical, regulatory and economic determinants of behaviour) components are crucial when considering lifestyle and prevention strategies and their successful implementation. As a complementary strategy, personalised prevention, which aims to prevent the onset, the progression and recurrence of diseases, has been the focus of a recently funded project (64), as well as a recent Horizon Europe RIA call (see Table 1).

However, it should be considered that even when public health policies at the national level tackle prevention, they do not necessarily integrate or consider the most updated scientific evidence due to a wide range of contextual factors and pressures (65).

### 4.3 Raising public awareness and improving education and training of healthcare actors (linked to RPIs 2, 3, 11)

In light of the importance of prevention to mitigate disease prevalence and reduce morbidity and mortality, a framework for patients, clinicians and the general public should be supported in order for them to make the necessary behavioural changes and be aware of any environmental risks. Patients’ lifestyle factors should be considered in medical consultations, avoiding immediately prescribing medications without first considering possible lifestyle interventions (66). As mentioned above, these require behavioural changes; specific initiatives should be put in place to implement citizen and patient engagement on topics such as primary prevention. This would also help understanding and overcoming vaccine hesitancy (67). In addition, few stakeholders have expressed some concerns about the lack of awareness among all the involved key actors, including teachers, health practitioners, and policymakers. For instance, in medical schools, only 25–30% of future physicians receive nutrition information or can provide practical nutritional advice (68). Even though the evidence of its impact on behaviour is limited, education on lifestyle and prevention, or in other words, health literacy (69), should ideally start already at primary school level. This should be combined with improvements in school climate (less stress, fear and bullying), school environments (safe, calm, without vending machines) and school policies (rules about substance use and antisocial behaviours), to facilitate and sustain behavioural changes, increase quality of life and reduce the risk of chronic diseases later on. Such two-pronged prevention approaches that focus both on improving individual protective behaviour and on the systemic drivers for potentially harmful behaviour can address additional societal challenges, such as social inequity and climate change at school level.

Another critical aspect is that medical practitioners’ education is traditionally focused on a single disease, whilst multi-morbidities and person-centred care should be better accounted for.

Furthermore, it would be essential to involve educators and patients’ associations in this discussion, to incorporate a new mindset in primary healthcare. Understanding patients’ experience and integrate this knowledge through all the steps of drug development and the implementation process should be considered a priority; some recent EC calls for proposals have addressed this important aspect (see Table 1).

Moreover, training courses addressed to clinicians and scientists on how to properly design clinical research could be considered. AI technologies could be leveraged to improve general data literacy in the coming decades, improving understanding of the multidimensionality of common morbidities among citizens and patients.

### 4.4 The impact of environmental pollution factors on human health (linked to RPIs 2, 3, 4)

Numerous survey respondents have identified environmental pollution as a public health concern warranting particular attention. In addition to being a well-established factor contributing to various diseases, as discussed in section 4.1, environmental pollution also significantly influences microbiota composition (70, 71). While there is growing awareness about air pollution, water pollution remains an underestimated or neglected carrier of environmental threats for diseases spreads, with direct consequences on human (and animal) health (72).

One in six deaths are due to pollution, accounting for 9 million deaths per year globally (73). Although there have been improvements in air and water pollution in households, ambient air pollution and hazardous chemical pollution is still on the rise. International alignment on policies and interventions will be key to decrease significantly the burden of pollution on human and other animal lives (74, 75). Some EU initiatives have the potential to drive evidence-based policies and funding for successfully addressing interlinked threats as pollution, climate change and health (see Table 1).

In addition, understanding the impact of environmental factors on disease onset is of pivotal relevance to designing suitable prevention strategies. This prompts the need to invest in tools and methods that allow us to have a better understanding of the effects of chemicals and other environmental factors on human health in order to enable sound decision making. Also, where research results clearly identify such effects, effective policies are needed to ensure people can live in healthy environments.

Nanomaterials and nano-plastics are also an environmental and health threat; their possibly detrimental impact on brain development and their associations with neurodegenerative diseases have been recently reported (76, 77).

In addition, a number of viral infections have been associated with NCDs, although with regional variations (78). However, when considering clinical studies, this kind of information is rarely available, or even when available and recorded in electronic health records, clinicians may not consider it due to lack of knowledge or inability to interpret data correctly. Tests to identify the possible presence of infections or other unsuspected factors, should be implemented to improve the quality of clinical studies, and AI should be leveraged to support clinicians’ decisions.

Recent large-scale EU public-public and public-private partnerships have been initiated to address existing gaps and challenges for health and care, including environment and health (see Table 1).

### 4.5 Improving clinical trial design, data sharing and data quality (linked to RPIs 5, 8, 9, 10)

Developing treatments, drugs or medical devices that are safe and effective for patients is critical. The path to a new treatment or therapy that has regulatory approval is long and complex, and too many conditions and diseases still remain poorly understood. According to survey results, the inappropriate interpretation of research results (e.g., due to poor or inappropriate study designs and settings and potentially leading to the identification of incorrect drug targets), suboptimal clinical trial design, inter-species differences, and the improper selection of animal models at the research or preclinical stage were considered the most relevant factors contributing to failure in drug development. These could contribute to failures at clinical trial stages or post-marketing drug withdrawal (e.g., for unintended toxic effects) (3, 15–17).

The design of clinical trials for drug testing has long been hindered by a lack of gender balance (79), and the poor inclusion of minority groups (80). Likewise, non-clinical research for drug discovery has largely used cell lines traditionally derived from Caucasian (often male) cells. This has translated over time into a massive bias on the results produced in the context of these activities, possibly contributing to later clinical trial failures (81). A recent IHI call addressed this issue (Table 1); further initiatives along this line are needed. Many studies derived from large biobanks (such as the UK Biobank (82)) often allow us to see only part of the big picture, which is particularly evident in research on ageing and age-related diseases (83). *In silico* technologies and AI will aid in better designing clinical trials, improving the heterogeneity of human cohorts, considering different ethnicities, gender balance, etc. (84).

Furthermore, research on vulnerable population groups/individuals (e.g., to food, alcohol, illicit drugs, etc.) is warranted to better understand gender differences in response to treatments or identify specific (epi) genetic backgrounds and disease susceptibilities (e.g., to diabetes, cardiovascular disease, cancer). In addition, assessing microbiome composition in childhood can help identify and predict possible predispositions to certain diseases later in life (85). Research on these topics and the creation of large data sets and clinical repositories should be supported, as it will both serve to build prevention policies and promote individual/personalised medicine approaches.

Programmes such as IHI have already taken steps to help improve the design of clinical trials and data quality (see Table 1), highlighting the need for multidisciplinary and multistakeholder approaches.

In addition, improving data sharing has been highlighted in the survey as one of the most effective policy interventions. Many IHI projects facilitate the sharing and curation of data from many different sources (see some examples in Table 1).

Notably, the widespread willingness to discuss data sharing and information transfer lacks a corresponding commitment to data curation, standardisation, and harmonisation, posing a significant challenge, given the substantial data generated in the health sector. In this context, the European Research Infrastructure for Biobanking & Biomolecular Resources (BBMRI-ERIC) offers a federated IT infrastructure across its hospital-integrated biobanks as single entry point to explore and access patient−/proband-consented samples, clinical phenotype data, as well as omics and imaging data in a quality-assured and GDPR conform manner (Table 1). However, the datafication process for the individual biobanks requires further support to fully leverage accessibility to samples and data.

In addition, DG RTD enforces the dissemination of the information and the publication of data, further boosted by the integration of SMEs in consortia; some recent calls on data-driven approaches are listed in Table 1.

In the era of big data, re-usability of curated data is another crucial aspect; the potential of AI and machine learning can only be realised with high-quality data to feed the systems, and greater efforts should be made to ensure data quality and accessibility (e.g., data FAIR-ification).

Moreover, there is the need to support institutions and repositories for the curation and maintenance of data after projects have finished, to reuse valuable data, avoiding redundant experiments, and preventing unnecessary expenditure of time and resources.

In the health sector, when considering AI applications or data sharing, restrictions to data access are incredibly high, and the integration of personal/patient clinical data requires an additional level of curation efforts. The EC, through the most recent *EU Digital Act* (86) and the *European Health Data Space regulation* (87), considers the possible legal aspects associated with data access.

Additional important initiatives have been put in place by the European Medicines Agency (EMA) and the Heads of Medicines Agencies (HMA) to implement overarching knowledge sharing and create a knowledge ecosystem (see Table 1).

When considering data quality, editorial boards of peer-reviewed journals also play an important role. Critical assessment by reviewers of manuscripts submitted to peer-reviewed journals would help reduce the need to critically appraise studies after publication, a task that can be very expensive and time-consuming, especially for small companies. Therefore, editors should critically select their reviewers when sending manuscripts for review.

In addition, concerning publication and data sharing, findings that contradict original research hypotheses, previous evidence or predictions (the so called ‘negative results’) are not or very rarely are published. This does not help identify, e.g., unsuitable (drug) targets or biomarkers of disease diagnosis and progression, or research discoveries that did not move to the clinical level. Knowing contradictory results would help reduce (translational) failures and help mitigate the reproducibility crisis (88). This would also require the development of innovative/alternative publishing models (some examples are provided in Table 1), or innovative approaches to managing data, with the creation of repositories to store and curate data for the long term.

In addition, due consideration should be given to instances where initially published results are later found to be inaccurate. However, it has been recognised by some stakeholders that there are no real incentives for researchers to change models or research strategies when needed, or to look critically at what other researchers are working on, because this is considered time consuming and not publishable. This prompts the need to build heterogeneous, multidisciplinary and multisectoral research communities (see section 4.7 for further insights on this aspect).

### 4.6 Supporting innovation in biomedical research (linked to RPIs 5, 7, 8)

Survey results indicate that future calls for proposals and funding programmes should give priority to human-based methodological approaches focused, e.g., on the use of human cohorts, samples and data, the development and application of complex human-based models, and innovative *in silico* technologies, in an effort to maximise societal impact of funded research. These approaches are already (and will continue) opening the door to personalised medicine, representing valuable alternatives to traditional, overly simplistic *in vitro* test systems or animal models, often poor predictors of human physiology, development, immunity and metabolism (89–94).

Only 4% of survey respondents think that research based on animals or animal-derived materials is conducive to societal impact; however, judging from the number of animals used in research (95), a significant part of the scientific community considers that the use of animals is needed to mimic biology/disease complexity, apart from remaining a mandatory requirement for the assessment of chemical and drug safety. However, approaches aiming at reducing, refining, and ultimately replacing the use of animals are routinely implemented in regulatory guidelines wherever applicable, as also stated in the *EMA Guidelines on the principles of regulatory acceptance of 3Rs* (96). The application of human-relevant technologies, encompassing complex *in vitro* models and *in silico* technologies, is becoming progressively more important in both basic and applied biomedical research to explore a large variety of fundamental research aspects [for instance, to model host-microbiome interactions in disease onset (97)], with the potential to inform prevention research strategies.

As commented in section 4.5, clinical cohort design can be challenging and undermined by several issues, such as, but not limited to, gender/age/ethnicity imbalance, incorrect patient stratification, neglecting comorbidities, etc. Innovative technologies, such as *in silico* trials (98, 99), AI and machine learning are already enabling working on clinical cohorts, improving their design (84), supporting drug discovery and development (100), as well as drug repurposing, helping improve overall productivity, while mitigating clinical trials’ burden and reducing time (100, 101). Using *in silico* tools has scalability potential that could not be conceivable with other technologies. On the other hand, human-derived models with high clinical mimicry (102) and high-throughput screening platforms based on human cells (103), support drug discovery at the non-clinical stage, reducing time, labour, and costs.

Digitisation of healthcare systems and digital technologies (e.g., applications to measure a wide range of different parameters, such as blood pressure, sleeping rate, food intake, etc.) and activities related to digitisation are currently being conducted in basically all health areas (104). A lot of knowledge is embedded in these technologies, and the sharing of this knowledge to technical and non-technical communities is increasing exponentially. In addition, improving accessibility to biobanks and data set repositories, with individual set-ups, could enable personalised medicine approaches (105).

The rising demand for dependable human-centred methodologies and the escalating ethical considerations regarding animal usage in biomedical science and regulatory testing have spurred numerous research initiatives and the initiation of several projects at EU level (some remarkable examples are reported in Table 1).

Notably, 27% of survey participants viewed allocating more funding to research projects focused on innovative animal models (e.g., humanised mouse models) as ‘ineffective,’ whilst 34% deemed it a ‘somewhat effective’ policy intervention. These contradictory responses further underscore the dilemma in using animal models for disease and safety studies. Despite progress and investments in developing innovative human-based (non-animal) methods and models, further investment is needed, particularly to support their validation and benchmarking, assessing their readiness level. This will help increase confidence in their use, enabling decision-making and the regulatory approval of new drug candidates tested using new models (106). This represents a top priority, considering that many suitable methods have been developed over the past decade, but most of them have not been validated and implemented in industrial settings. Moreover, even when valid methods and technologies are available, they are often not utilised [e.g., to test chemical safety (107)]. In general, more effort and funding are needed to support implementation science. Aside from their use in drug development, also the clinical utility of new approaches needs to be further assessed; this has been addressed in a recent IHI funding call (Table 1).

Another aspect that was emphasised during the roundtable discussion, is the lack of investment into understanding the barriers that prevent the uptake and use of existing knowledge and innovative tools. The adoption of any innovative approach into regulations is not an easy process, and every step of the development of a new technology or product is subjected to a lengthy regulatory approval workflow, especially in the field of *in silico* medicine or organ-on-chip technologies. To help companies adopt these technologies, this process should be streamlined to be made more efficient and reduce inefficiencies, for example, by involving regulators earlier in the technology development process. This could speed up their wider adoption and encourage more R&D activities. Some recent initiatives at EU level are in line with these principles (Table 1).

### 4.7 Additional aspects considered during the roundtable discussion: impact of funded research and the role of interdisciplinary approaches (linked to RPIs 4–9)

While funding initiatives are intended to foster societal impact, many scientists often lack the time and resources to strategise follow-up plans for translating their research findings into practical, impactful solutions for society. This phenomenon frequently confines significant breakthroughs to the realm of peer-reviewed publications, serving primarily as stepping stones for securing further funding rather than effecting tangible improvements in public health. New approaches and innovative funding mechanisms should be considered, with the aim to move the research community away from publishing to secure the next grant, toward a system that rewards true innovation and impact on patients and society. Enhancing and broadening connections between academia, industry, policymakers, and regulatory bodies would streamline and enhance the utilisation and valorisation of research outcomes. Along this line, Horizon Europe has been and is supporting impact-driven, multidisciplinary, translational research. Most recent EC funding calls require a step-by-step assessment of the envisioned short-, medium- and long-term impacts of a research project, asking proponents to explain what consequences their research could have at societal level, and also how they plan to interact with the regulatory agencies (see some examples in Table 1).

Lack of translatability of scientific results could be associated with multiple reasons, including (i) the selection of unreliable methodological approaches and experimental preclinical models (17), (ii) not considering real-world data when planning research settings and conditions (108), or (iii) lack of follow-up funding (36). In addition, lack of knowledge or misconception of disease mechanisms may lead to the selection of wrong druggable targets (109). Altogether, this has resulted in thousands of identified biomarkers and targets proven unsuitable in clinical practice [e.g., (110)]. Initiatives focused on the validation of biomarkers (e.g., the IHI call reported in Table 1) should be put in place to support the validation and qualification of newly discovered biomarkers.

The query for funding entities lies in devising strategies to incentivise research projects capable of yielding outcomes that can be effectively implemented within the healthcare system and the market. In line with this, under H2020 and Horizon Europe (111), there is a notable emphasis on advancing research, at least in part, up to the Technology Readiness Level (TRL) ladder to enhance the marketability of the research outcomes. This is also part of the European Innovation Council (EIC) (112) and the European Institute of Innovation & Technology (EIT) (113) mandates.

As a general observation, research proposals should be evaluated on their successful outcomes and plans for translating discoveries into actions, rather than their intentions. Retrospective assessment of funded research can help identify factors for success and acknowledge that societal impact may take time to become evident (36).

Important initiatives aimed at monitoring the broader impact of funded research have been undertaken by the EC during the preparatory phase of Horizon Europe in addition, robust and reliable indicators to monitor the retrospective impact of EU-funded research should be developed and implemented (Table 1). Fostering multidisciplinarity through partnerships between academic researchers (including social scientists), technology developers, industry, policymakers and regulators can help streamline the application and maximise the impact of research outputs. Interdisciplinarity is entrenched in the research projects funded by DG RTD and IHI (Table 1). Assessing the genuine value of multidisciplinary approaches that go beyond the cumulative contributions of individual disciplines examining the same research question through a singular lens, can be challenging. Establishing spaces and platforms to facilitate community bridging in a multi/interdisciplinary way (from the design of the projects to their later implementation) will help identify the right individuals. Interdisciplinarity is also relevant when planning clinical trial cohorts, considering potential exposure to environmental chemicals or environmental hazards, triggered disease pathways distinguishing patient cohorts, and disease heterogeneity and exposure scenarios.

In addition, integrating knowledge and data about chemicals’ effects on the environment and human health, in line with the *Triple Planetary Crisis* concept (encompassing climate change, pollution, and biodiversity loss) (114) can help improve current chemical management at the regulatory level, reducing costs and time.

The EU framework programmes have evolved to emphasise societal challenges and Sustainable Development Goals, incorporating innovative funding instruments for directionality, stakeholder participation, and experimentation (115).

## 5 Conclusion and actionable recommendations

Survey results and stakeholder opinions collected during the online roundtable discussion emphasised that mental health disorders, metabolic syndrome, cancer, AMR, cardiovascular diseases, aging-related conditions, neurodegenerative diseases (e.g., Alzheimer’s), environmental pollution, its implications on diseases like cardiovascular diseases, metabolic syndromes, and cancer, as well as the influence of climate change on infectious vector-borne diseases, are the most urgent public health challenges.

Furthermore, early disease detection and diagnosis, primary prevention, the influence of environmental factors on disease risk, personalised medicine approaches, improved access to care and therapies, maintaining patients’ quality of life, and addressing medication shortages for specific groups, particularly rare diseases, children, and pregnant women, were identified as key areas deserving special attention as UMNs.

The persistence of these well-known public health challenges and UMNs as priorities for various stakeholders highlights that, despite ongoing investments, significant challenges remain. Addressing these issues requires continued long-term investments and efforts. Overall these findings could help identify research areas deserving prioritisation in the EU research policy agenda.

Biomedical research focusing on the aforementioned key public health topics and UMNs can benefit from interdisciplinary collaborations. At the EU level, various initiatives, research programs and funding opportunities have been launched to foster multidisciplinarity, promote stakeholder engagement, broaden the societal impact of biomedical research, and ensure the sustainability of research outcomes.

Ultimately, translating knowledge into policies generally takes time, involving the summarisation of data and distillation into regulations. Research proposals should be carefully crafted to ensure that their findings can be effectively converted into actionable policies. To achieve this, research should be designed with consideration for the translational relevance of methods and models, considering the context of use and how relevant these models are to reply to research questions. In addition, how research outputs will lead to tangible short, medium and long-term impacts should be carefully planned, and retrospective assessment of such impacts should be undertaken by means of reliable and robust indicators.

The 33 actionable recommendations reported below, organised into eight main topics (also detailed in Table 1), have been formulated based on survey findings and feedback gathered during the multi-stakeholder roundtable discussion. These recommendations aim to enhance the translatability of public health and biomedical research outputs and their implementation into transformative policies.

**Public health challenges and UMNs warranting prioritisation**

- R1: Prioritise research on primary prevention strategies to reduce the burden of NCDs and associated comorbidities.
- R2: Dedicated calls for proposals should be initiated to investigate the impact of environmental pollution on disease onset.
- R3: Encourage participative collaboration with low-middle income countries to effectively address emerging public health threats and environmental concerns.
- R4: Prioritise initiatives addressing the lack of medicines for rare diseases, children, and pregnant women.

**Prevention and early diagnosis**

- R5: Efforts to improve early detection of disease among women should be put in place.
- R6: Develop additional tools and mechanisms to demonstrate the economic benefits of prevention.
- R7: MS should implement prevention strategies based on the latest scientific evidence and ensure accountability.
- R8: Allocate more funding on personalised prevention strategies to prevent the onset, progression, and recurrence of diseases

**Public awareness, education and training of healthcare actors**

- R9: Include comprehensive nutrition education in medical school curricula to ensure that future physicians are equipped with the knowledge and skills to provide practical nutritional advice.
- R10: Improve individual protective behaviour and address systemic drivers of harmful behaviour to tackle societal challenges.
- R11: Reform medical practitioners’ education to include training that addresses multi-morbidities and emphasises person-centred care.
- R12: Involve educators, patients and patient associations to integrate their view into primary healthcare.

**Impact of environmental pollution on health**

- R13: Align international policies and interventions to reduce the burden of pollution on humans and animals, and ensure clean and healthy environments for all.
- R14: Invest in the development of tools and methods to better understand and address the impact of chemicals, viral infections, and other environmental factors on human health.

**Clinical trial design**

- R15: Increase support for clinical studies addressing equitable access and inclusivity and leveraging AI technologies.
- R16: Develop training courses for clinicians and scientists on proper clinical research design.
- R17: Support research on vulnerable populations to understand gender differences in treatment responses and identify (epi) genetic disease susceptibilities.

**Data sharing and data quality**

- R18: Increase efforts to promote the curation, standardisation, and harmonisation of health data.
- R19: Provide support for the datafication process of individual biobanks to enhance accessibility to samples and data across the EU.
- R20: Allocate a significant portion of new funding to projects that emphasise data reusability and build on previously released data.
- R21: Reduce data access restrictions in the health sector for AI applications and data sharing.
- R22: Enhance curation efforts for integrating personal and patient clinical data.
- R23: Editors should rigorously select reviewers for peer-reviewed journals to ensure critical assessment of manuscripts.
- R24: Incentivise publication and data sharing of findings that contradict original research hypotheses or previous evidence.

**Supporting innovative biomedical research**

- R25: Allocate funds to validate and benchmark innovative models and methods to increase confidence in their use and support implementation science.
- R26: Enhance accessibility to biobanks and data repositories, allowing for individualised setups, to facilitate the adoption of personalised medicine approaches.
- R27: Further assess the clinical utility of new approaches with dedicated funding.
- R28: Streamline the regulatory approval process for AI technologies by involving regulators earlier in the technology development process.

**Research impact and multidisciplinarity**

- R29: Foster continuing partnerships between academia, industry, policymakers, and regulatory bodies to streamline the application and maximise the impact of research outputs.
- R30: Establish spaces and platforms to facilitate community bridging in a multi/interdisciplinary manner from project design to implementation.
- R31: Implement further initiatives to support the validation and qualification of newly discovered biomarkers.
- R32: Research projects with clear potential for practical implementation in the healthcare system and market should deserve prioritisation.
- R33: Robust and reliable indicators should be developed and implemented to monitor the retrospective impact of funded research.

## Author contributions

FPi: Conceptualization, Data curation, Formal analysis, Methodology, Writing – original draft, Writing – review & editing. GB: Writing – review & editing. PD: Writing – review & editing. CB: Writing – review & editing. SV: Writing – review & editing. LE: Writing – review & editing. A-CF: Writing – review & editing. LF: Writing – review & editing. AG: Writing – review & editing. CG: Writing – review & editing. JH: Writing – review & editing. IH: Writing – review & editing. CK: Writing – review & editing. UL: Writing – review & editing. PL: Writing – review & editing. HL: Writing – review & editing. BM: Writing – review & editing. MM: Writing – review & editing. FPa: Writing – review & editing. RP: Writing – review & editing. VR: Writing – review & editing. ER: Writing – review & editing. HC: Supervision, Writing – review & editing.
