# Supplementary material for: What public health challenges and unmet medical needs would benefit from interdisciplinary collaboration in the EU? A survey and multi-stakeholder debate
Source: Front Public Health. 2024 Jul 22;12:1417684. doi: 10.3389/fpubh.2024.1417684 (PMC11298480; doi:10.3389/fpubh.2024.1417684)
Supplement: Supplementary file 1 [file Data_Sheet_1.PDF]

Public health priorities and unmet biomedical needs - Have your say!

Fields marked with * are mandatory.

Introduction

Humane Society International (HSI) Europe has launched the following survey aimed to gather feedbacks and opinions on what public health challenges and unmet biomedical needs deserve well-planned funding strategies and prioritization at EU level.
Understanding the possible factors underlying today and emerging public health challenges is essential to plan bold, innovative and effective research and policy intervention strategies.

Survey results will be published in a peer-reviewed article, along with multi-stakeholder recommendations gathered during a roundtable recently organized by HSI/Europe.

Be aware that your contribution will remain anonymized.

Feel free to share the link to the survey to representatives of public health or research organizations who you believe could be interested in these topics.

Privacy statement
[HSI privacy policy]

- [ ] I have read and accept the terms of the privacy statement

General Details

* Your primary affiliation category

- ○ Academia
- ○ Contract research organization
- ○ EU institutions
- ○ Governmental/public
- ○ Health care (clinic or hospital)
- ○ Industry
- ○ Non Governmental Organisation
- ○ Other

* If 'other', specify type of affiliation:

* What is your area of work?

at least 1 choice(s)

- [ ] Clinic
- [ ] Education/communication
- [ ] Epidemiology
- [ ] Prevention
- [ ] Public health/health care
- [ ] Regulatory sector
- [ ] Research (basic or applied)
- [ ] Research funding
- [ ] Science and Research Policy
- [ ] Other

* If 'other', specify area of work:

* What is your primary role?

at least 1 choice(s)

- [ ] Data analyst
- [ ] Educator
- [ ] Group leader/coordinator
- [ ] Medical practitioner
- [ ] Patients' representative
- [ ] Regulatory decision-maker
- [ ] Senior scientist/investigator
- [ ] Administrator
- [ ] Other

* If 'other', specify your primary role:

* What is the primary country where you work?

- ( ) AF - Afghanistan
- ( ) AL - Albania
- ( ) DZ - Algeria
- ( ) AD - Andorra
- ( ) AO - Angola
- ( ) AG - Antigua and Barbuda
- ( ) AR - Argentina
- ( ) AM - Armenia
- ( ) AU - Australia
- ( ) AT - Austria
- ( ) AZ - Azerbaijan
- ( ) BS - Bahamas

- BH - Bahrain
- BD - Bangladesh
- BB - Barbados
- BY - Belarus
- BE - Belgium
- BZ - Belize
- BJ - Benin
- BT - Bhutan
- BO - Bolivia
- BA - Bosnia and Herzegovina
- BW - Botswana
- BR - Brazil
- BN - Brunei Darussalam
- BG - Bulgaria
- BF - Burkina Faso
- BI - Burundi
- CV - Cabo Verde
- KH - Cambodia
- CM - Cameroon
- CA - Canada
- CF - Central African Republic
- TD - Chad
- CL - Chile
- CN - China
- CO - Colombia
- KM - Comoros
- CG - Congo
- CR - Costa Rica
- CI - Côte D'Ivoire
- HR - Croatia
- CU - Cuba
- CY - Cyprus
- CZ - Czechia
- CD - Democratic Republic of the Congo
- DK - Denmark
- DJ - Djibouti
- DM - Dominica
- DO - Dominican Republic
- EC - Ecuador
- EG - Egypt
- SV - El Salvador
- GQ - Equatorial Guinea
- ER - Eritrea
- EE - Estonia
- SZ - Eswatini
- ET - Ethiopia
- FJ - Fiji

- FI - Finland
- FR - France
- GA - Gabon
- GM - Gambia
- GE - Georgia
- DE - Germany
- GH - Ghana
- GR - Greece
- GD - Grenada
- GT - Guatemala
- GN - Guinea
- GW - Guinea Bissau
- GY - Guyana
- HT - Haiti
- HN - Honduras
- HU - Hungary
- IS - Iceland
- IN - India
- ID - Indonesia
- IR - Iran
- IQ - Iraq
- IE - Ireland
- IL - Israel
- IT - Italy
- JM - Jamaica
- JP - Japan
- JO - Jordan
- KZ - Kazakhstan
- KE - Kenya
- KI - Kiribati
- KW - Kuwait
- KG - Kyrgyzstan
- LA - Laos
- LV - Latvia
- LB - Lebanon
- LS - Lesotho
- LR - Liberia
- LY - Libya
- LI - Liechtenstein
- LT - Lithuania
- LU - Luxembourg
- MG - Madagascar
- MW - Malawi
- MY - Malaysia
- MV - Maldives
- ML - Mali
- MT - Malta

- MH - Marshall Islands
- MR - Mauritania
- MU - Mauritius
- MX - Mexico
- FM - Micronesia
- MC - Monaco
- MN - Mongolia
- ME - Montenegro
- MA - Morocco
- MZ - Mozambique
- MM - Myanmar
- NA - Namibia
- NR - Nauru
- NP - Nepal
- NL - Netherlands
- NZ - New Zealand
- NI - Nicaragua
- NE - Niger
- NG - Nigeria
- KP - North Korea
- MK - North Macedonia
- NO - Norway
- OM - Oman
- PK - Pakistan
- PW - Palau
- PA - Panama
- PG - Papua New Guinea
- PY - Paraguay
- PE - Peru
- PH - Philippines
- PL - Poland
- PT - Portugal
- QA - Qatar
- MD - Republic of Moldova
- RO - Romania
- RU - Russian Federation
- RW - Rwanda
- KN - Saint Kitts and Nevis
- LC - Saint Lucia
- VC - Saint Vincent and the Grenadines
- WS - Samoa
- SM - San Marino
- ST - Sao Tome and Principe
- SA - Saudi Arabia
- SN - Senegal
- RS - Serbia
- SC - Seychelles

- SL - Sierra Leone
- SG - Singapore
- SK - Slovakia
- SI - Slovenia
- SB - Solomon Islands
- SO - Somalia
- ZA - South Africa
- KR - South Korea
- SS - South Sudan
- ES - Spain
- LK - Sri Lanka
- SD - Sudan
- SR - Suriname
- SE - Sweden
- CH - Switzerland
- SY - Syrian Arab Republic
- TJ - Tajikistan
- TZ - Tanzania
- TH - Thailand
- TL - Timor-Leste
- TG - Togo
- TO - Tonga
- TT - Trinidad and Tobago
- TN - Tunisia
- TR - Turkey
- TM - Turkmenistan
- TV - Tuvalu
- UG - Uganda
- UA - Ukraine
- AE - United Arab Emirates
- GB - United Kingdom
- US - United States of America
- UY - Uruguay
- UZ - Uzbekistan
- VU - Vanuatu
- VE - Venezuela
- VN - Viet Nam
- YE - Yemen
- ZM - Zambia
- ZW - Zimbabwe

Survey

* Q1: In your opinion, what are the most urgent public health challenges today?

between 1 and 5 choices

- [ ] Mental health disorders (e.g., dementia, depression, anxiety, schizophrenia, eating disorders, etc.

- [ ] Developmental disorders
- [ ] Cancer
- [ ] Metabolic syndrome diseases (e.g., diabetes, obesity, hypertension)
- [ ] Cardiovascular diseases
- [ ] Allergies and airways/respiratory diseases
- [ ] Autoimmune disorders
- [ ] Infectious diseases
- [ ] Substance abuse disorders
- [ ] Malnutrition and food safety
- [ ] Sexual and reproductive health challenges
- [ ] Environmental pollution
- [ ] Anti-microbial resistance (AMR)
- [ ] Other

* If 'other', specify public health challenge(s):

* Q2: In your opinion, what are the most relevant unmet (bio)medical needs that deserve prioritization in the research and policy agenda (at member states and EU level)?

between 1 and 5 choices

- [ ] Better preserving quality of life of those affected by (non-communicable) diseases
- [ ] Early detection/diagnosis (e.g., identification of early biomarkers, screening and secondary prevention) of some diseases
- [ ] Halt progression of disease
- [ ] Personalized medicine approaches
- [ ] Need for effective treatments that do not have side effects for some diseases
- [ ] Manage the burden of treatments used for disease
- [ ] Incentivize research on rare (or ultra-rare) diseases
- [ ] Increase investment on primary prevention to reduce disease risk
- [ ] Better inclusion of ethnic minority groups, different socio-economic background/education and genders in research
- [ ] Better involvement of patients (associations) in medicines development process
- [ ] Better access to care and therapies
- [ ] Better understanding of comorbidities
- [ ] Better understanding the impact of environmental factors in the onset of diseases
- [ ] Research and development on digital health and real-world data
- [ ] Other

* If 'other', specify unmet (bio)medical need:

Q3: To date, the prevalence and incidence of many of the aforementioned diseases remain high. Several factors may contribute to this problem. Can you rate their relevance?

	Irrelevant	Somehow relevant	Relevant	Highly relevant	Not sure
* Insufficient (or lack of) research funding in specific disease areas	○	○	○	○	○
* Inappropriate design of Calls for Proposals	○	○	○	○	○
* Inappropriate design of funded biomed research proposals (e.g., poor methodological approach, ineffective partnerships, inappropriate timing, etc.)	○	○	○	○	○
* Lack of knowledge about novel or emerging contributing risk factors	○	○	○	○	○
* Low public awareness about already known risk factors (e.g., inefficient knowledge dissemination and public outreach)	○	○	○	○	○
* Insufficient investment on disease etiology and/or epidemiology research	○	○	○	○	○
* Insufficient investment on primary prevention research	○	○	○	○	○
* Inefficient implementation of known prevention measures	○	○	○	○	○
* Drug failure or lack of effective drugs for some diseases	○	○	○	○	○

If needed, specify other contributing factor(s):

* Q4: What specific research activities do you think deserve more investment/resources to better face the aforementioned public health challenges and unmet medical needs?

between 1 and 3 choices

- ☐ Basic and applied biomedical research (e.g., to investigate novel disease mechanisms and identify new druggable targets)
- ☐ Education/training and dissemination (e.g., educational or public outreach activities)
- ☐ Etiology and epidemiology research (e.g., to investigate still unknown risk factors)
- ☐ Preclinical and clinical research (e.g., to test pharmacological or non-pharmacological interventions)
- ☐ Primary prevention research (e.g., through observational or intervention studies)
- ☐ Secondary prevention research (e.g., to design or implement the use of novel diagnostic and screening tools /devices)
- ☐ Other

* If 'other', specify research activity(ies):

* Q5: Concerning research design, in your opinion what are the approaches/tools that could be more effective to gain research translational success and ultimately public health impact?

- [ ] Human cohorts, human samples or data sets
- [ ] Innovative human-based models (e.g., complex cellular/tissue/organ models)
- [ ] *In silico* tools (e.g., artificial intelligence, machine learning)
- [ ] Live animals (e.g., transgenic/humanized animals) or animal-derived materials
- [ ] No opinion (e.g., it depends on the research question/context of use)
- [ ] Other

* If 'other', specify approach(es):

Q6: Concerning drug attrition, several factors may contribute to failures in drug development. Can you rate their relevance?

	Irrelevant	Somehow relevant	Relevant	Highly relevant	Not sure
* Inappropriate selection of *in vivo* (animal) models at research or preclinical stage	○	○	○	○	○
* Inappropriate selection of *in vitro* (animal- or human-derived) models at research or preclinical stage	○	○	○	○	○
* Wrong or inappropriate pharmacological (or non-pharmacological) target selection	○	○	○	○	○
* Inappropriate clinical trial design (e.g., biased selection or low number of participants)	○	○	○	○	○
* Neglecting important aspects (e.g., pharmacokinetic/pharmacodynamic, off-target effects, etc.)	○	○	○	○	○
* Inappropriate interpretation of research results	○	○	○	○	○
* Inter-species differences responsible for lack of efficacy or toxicity issues	○	○	○	○	○

If needed, specify what other important factor(s) may contribute to drug failure

Q7: Some policy interventions at EU or member state level could be envisaged to tackle public health emerging/unsolved challenges or other unmet biomedical needs. Could you rate their effectiveness?

	Not effective	Somehow effective	Effective	Most effective	Not sure
* Increase funding on basic-applied research (e.g., to identify novel disease mechanisms)					
* Increase funding on identification of novel etiological factors (e.g., environmental or genetic risk factors)					
* Increase funding on biomed projects focused on innovative human-based technologies and their validation					
* Improve research institutes' access to human biobanks					
* Incentivize sharing of big data and clinical trial information					
* Increase funding on biomed projects focused on innovative animal models (e.g., humanized mouse models)					
* Increase funding on primary prevention research (e.g., observational and intervention studies in human cohorts)					
* Increase funding on public awareness and education (e.g., to improve knowledge on prevention)					
* Incentivize dialogue with patients' associations about unmet medical needs and research priorities					
* Increase funding on drug repurposing					
* Increase funding on new drug discovery					

If needed, specify what other important intervention(s) should be considered

Availability

* As a follow-up of your contribution to this survey, are you willing to be contacted again for future activities?

- Yes (please provide your contact details below)
- No

* First and last name

* Email

Thank you for your participation!
