# Supplementary material for: What public health challenges and unmet medical needs would benefit from interdisciplinary collaboration in the EU? A survey and multi-stakeholder debate
Source: Front Public Health. 2024 Jul 22;12:1417684. doi: 10.3389/fpubh.2024.1417684 (PMC11298480; doi:10.3389/fpubh.2024.1417684)
Supplement: Supplementary file 2 [file Data_Sheet_2.docx]

Supplementary Material

What public health challenges and unmet medical needs would benefit from interdisciplinary collaboration in the EU? A survey and multi-stakeholder debate

Francesca Pistollato*, Gregor Burkhart, Pierre Deceuninck, Camilla Bernasconi, Sergio Di Virgilio, Luca Emili, Anne-Charlotte Fauvel, Luisa Ferreira Bastos, Annalisa Gastaldello, Chiara Gerardi, Jens K. Habermann, Ioan Hanes, Christina Kyriakopoulou, Uma Lanka, Paolo Lauriola, Hugh Laverty, Benoit G.C. Maisonneuve, Milena Mennecozzi, Francesco Pappalardo, Roberta Pastorino, Vilma Radvilaite, Erwin L. Roggen and Helder Constantino

*** Correspondence:** Francesca Pistollato: [fpistollato@hsi.org](mailto:fpistollato@hsi.org)

# Methodological approach used to disseminate the survey via email

A simple and reproducible approach for using PubMed to generate large email lists of potential participants was used, as previously described (Khalifa M. Stud Health Technol Inform. 2019 Jul 4:262:348-351. available at <https://pubmed.ncbi.nlm.nih.gov/31349339/>).

A first targeted group of emails was sent considering emails retrieved using PubMed on January 5^th^, 2024, considering as filters only English papers published in the last 5 years and the following combinations of search terms and Boolean operators:

(European union[Title/Abstract] AND unmet medical need[Title/Abstract] OR public health[Title/Abstract] OR biomedical research[Title/Abstract] OR research impact[Title/Abstract]) AND (European union[Affiliation] AND unmet medical need[Affiliation] OR public health[Affiliation] OR biomedical research[Affiliation] OR research impact[Affiliation]).

From the generated list of emails, only those with an EU-related (or international) affiliation were retained, and a total of 1,426 emails were sent.

A second targeted group of emails was sent considering emails retrieved using PubMed on January 10^th^, 2024, considering as filters only English papers published in the last 1 year and the following combinations of search terms and Boolean operators:

(Europe[Affiliation] AND health policy[Affiliation] OR return on investment[Affiliation] OR non-animal methods[Affiliation] OR NAMs[Affiliation] OR animal models[Affiliation] OR health[Affiliation] OR drug failure[Affiliation] OR drug attrition[Affiliation] OR basic research[Affiliation] OR human-based[Affiliation] OR funding[Affiliation]) AND (Europe[Title/Abstract] AND health policy[Title/Abstract] OR return on investment[Title/Abstract] OR non-animal methods[Title/Abstract] OR NAMs[Title/Abstract] OR animal models[Title/Abstract] OR health[Title/Abstract] OR drug failure[Title/Abstract] OR drug attrition[Title/Abstract] OR basic research[Title/Abstract] OR human-based[Title/Abstract] OR funding[Title/Abstract])

From the generated list of emails, only those with an EU-related (or international) affiliation were retained, and a total of 1,345 emails were sent.

# Survey results: additional comments provided by survey respondents in reply to some of the survey questions

The following is a summary of additional comments provided by survey respondents in reply to some of survey questions Q1-Q7 (under “others”).

In reply to Q1 *(In your opinion, what are the most urgent public health challenges today?*), among the “other” challenges a few respondents indicated:

- ageing and neurodegenerative diseases (note: although dementia was already included among “mental health disorders”),
- social determinants of health,
- climate change,
- early identification of genetic diseases,
- rare diseases.

In reply to *Q2 (In your opinion, what are the most relevant unmet (bio)medical needs that deserve prioritization in the research and policy agenda at member states and EU level?),* among the “other” unmet medical needs were indicated:

- psychosocial impact of disease,
- equity in health services,
- the need to complement traditional and alternative medicine,
- life course (environmental) exposures,
- commercial determinants of health,
- investing in novel mental health treatments,
- human-based research,
- education about disease prevention,
- the use of non-animal methods in drug development,
- the need for regulatory guidelines for SMEs,
- women's health (perimenopausal).

In reply to Q3 (*To date, the prevalence and incidence of many of the aforementioned diseases remain high. Several factors may contribute to this problem. Can you rate their relevance?),* under “other” some respondents indicated also:

- insufficient funding for screening genetic predispositions,
- an urgent call for increased investment in cardiac disease prevention,
- the need for greater involvement from patients and patient associations,
- active resistance to many public health actions by commercial interests,
- a noticeable gap in long-term investment for population-based fieldwork,
- insufficient research aimed at fostering health and normal resilience,
- insufficient attention paid to inequality and lack of resources,
- a general lack of awareness among the public and regulators about these critical issues,
- over-reliance on animal models for studying human diseases and inadequate adoption of human-centric innovative technologies in drug development and research,
- limited funding for developing new treatments,
- necessity to improve the access to biobanks and human biological samples, which is needed to develop personalised medicine approaches,
- no long-lasting efforts about research for a more personalised medicine,
- insufficient investment on monitoring technologies in real life,
- underinvestment in monitoring technologies,
- lack of integration within healthcare pathways,
- lack of focus on ‘One Health’ principle,
- inefficiencies in the co-creation process of innovative technologies between developers and end-users,
- blind spots in regular health checks,
- loss of knowledge regarding prevention measures across generations.

In reply to Q4 (*What specific research activities do you think deserve more investment/resources to better face the aforementioned public health challenges and unmet medical needs?*), among the 8% who selected “others”, here is a list of some of the recommendations provided:

- the need to translate research knowledge into behavioural changes and policies,
- research on social equity,
- more investment in traditional and conventional medicine,
- more funding on integrative medicine,
- more fieldwork for understanding diseases and disorders,
- the need to use modern techniques in population distribution studies,
- early detection of disease risks,
- the importance to consider human-relevant methods in research and drug development,
- the need to integrate public health data,
- the relevance of social science research,
- the need for large scale clinical trials in psychedelic therapies,
- the use of biomaterials and biosensors,
- the use of minimally invasive technologies,
- phage therapy for the treatment of bacterial infections,
- the need to remove regulatory barriers to in silico medicine,
- the need to prioritise artificial intelligence (AI)-supported research.

In reply to Q6 (*Concerning drug attrition, several factors may contribute to failures in drug development. Can you rate their relevance?*), in addition to the options provided, some participants also indicated:

- the lack of interest in low prevalence diseases,
- lack of interest in prevention research,
- the selection of inadequate study endpoints,
- the need to bridge the gap between research in the lab and clinical trials,
- insufficient interest in seeing the patient as a whole being and as a complex system in permanent interaction with their environment,
- the need to address human relevance and use human-based (rather than animal) models in drug discovery and development,
- the issue of research data reproducibility,
- inefficient regulatory frameworks and the need for better governance,
- inappropriate application of *in silico* trials and digital twin technologies in healthcare,
- inappropriate (public) data sharing,
- the unspecific use of drugs.

Finally, in reply to Q7 (*Some policy interventions at EU or member state level could be envisaged to tackle public health emerging/unsolved challenges or other unmet biomedical needs. Could you rate their effectiveness?*), among the “other” policy options, the following were suggested:

- increase funding on biomedical engineering translational/clinical projects,
- increase funding for implementing already existing knowledge (e.g., about known risk factors) into effective (behavior change) interventions,
- provide more funding for rare disease registries and post mortem diagnostic tools after sudden death in the young (autopsy, genetic testing),
- consider multi-morbidities (rather than individual disease) and the multi-factorial nature of disease, accounting for traditional, complementary and integrative healthcare (e.g., <https://www.tcih.org/>),
- improve complementarity of scope of funding instruments both at EU and Member State (MS) level,
- increase funding on biomedical projects focused on innovative human-based technologies and their validation,
- allocate more funding on health technological research,
- establish a network of European clinicians with expertise in high consequence infectious diseases and with access to a digital platform (as already done e.g., with the [European Reference Network (ERN) for rare diseases](https://www.eurordis.org/our-priorities/european-reference-networks/#:~:text=Eurordis%20%2D%20Rare%20Disease%20Europe&text=To%20address%20these%20challenges%2C%20the,travels%20rather%20than%20the%20patient.)),
- increase public awareness (and medical practitioners’ education) about primary prevention and lifestyle, ideally starting at primary school.

# Questions asked to participants of the online roundtable

At the online roundtable convened by HSI/Europe on November 24^th^ 2023, initial findings from the survey, encompassing responses from 79 participants, were shared. Subsequently, attendees were divided into three separate breakout sessions where they were invited to provide feedback on the preliminary survey results. Specifically, participants were prompted with the following questions:

PUBLIC HEALTH & UMNs:

Q1. Do you agree that these public health challenges (i.e., mental health diseases, metabolic syndrome, environmental pollution, cancer and AMR) and UMNs (i.e., primary prevention, early diagnosis/detection, environmental factors in disease risk, and personalised medicine) are the most relevant ones?

Q2. Why do they deserve prioritisation over other diseases or topics? What else should be prioritised?

AREAS NEEDING MORE FUNDING:

Q3. Do you agree that primary prevention implementation, risk factors identification, public awareness, basic/applied research deserve more investment to decrease disease prevalence/incidence?

Q4. Why do they deserve prioritisation over other topics? Or what else do you think should be prioritised?

Q5. Do you think past/present research initiatives/funding calls in the EU have adequately addressed these topics (see Q3)?

RESEARCH FUNDING & IMPACT:

Q6. Do you agree that future calls for proposals/funding programmes should give priority to research approaches focused on human cohorts/samples/data, human-based models, and in silico tools, to maximize societal impact of funded research?

Q7. Can you name any (past or current) EU research initiative/project/funding call that had/may have great potential of societal impact?

DRUG FAILURE:

Q8. Do you agree that these factors (i.e., inappropriate animal models, interspecies differences, wrong results’ interpretation, inappropriate clinical trial design) are the most relevant ones contributing to drug failure?

POLICY INTERVENTIONS:

Q9. Do you agree that these (i.e., improving data sharing; increasing public awareness; allocating funding on innovative human models; increasing funding on primary prevention; allocating funding on drug repurposing; allocating funding on basic/applied research; allocating funding on disease etiology; promoting dialogue with patients’ associations; improving access to biobanks) are the most effective policy interventions?

Q10. Any idea on how these policy interventions should/could be implemented?

Q11. Are you aware of any EU (or Member State) policy initiatives in line with these?

# Supplementary Figures

**
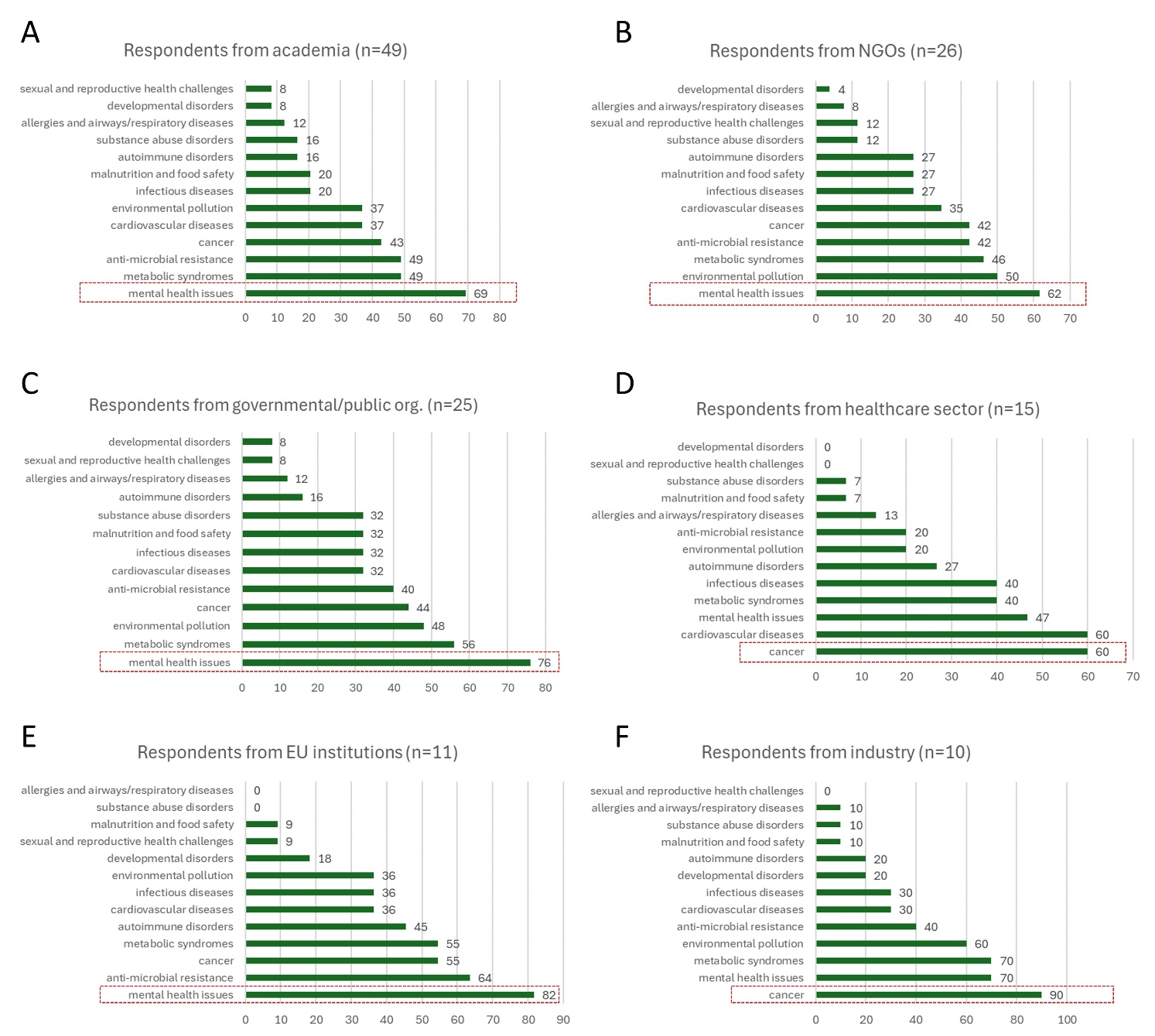
**

**Supplementary Figure 1.** Public health challenges’ ranking across different stakeholder groups: (A) academia, (B) NGOs, (C) government/public organisations, (D) healthcare, (E) EU institutions, and (F) industry. Participants were allowed to select between 1 and 5 options, which explains why the combined percentages exceed 100%.


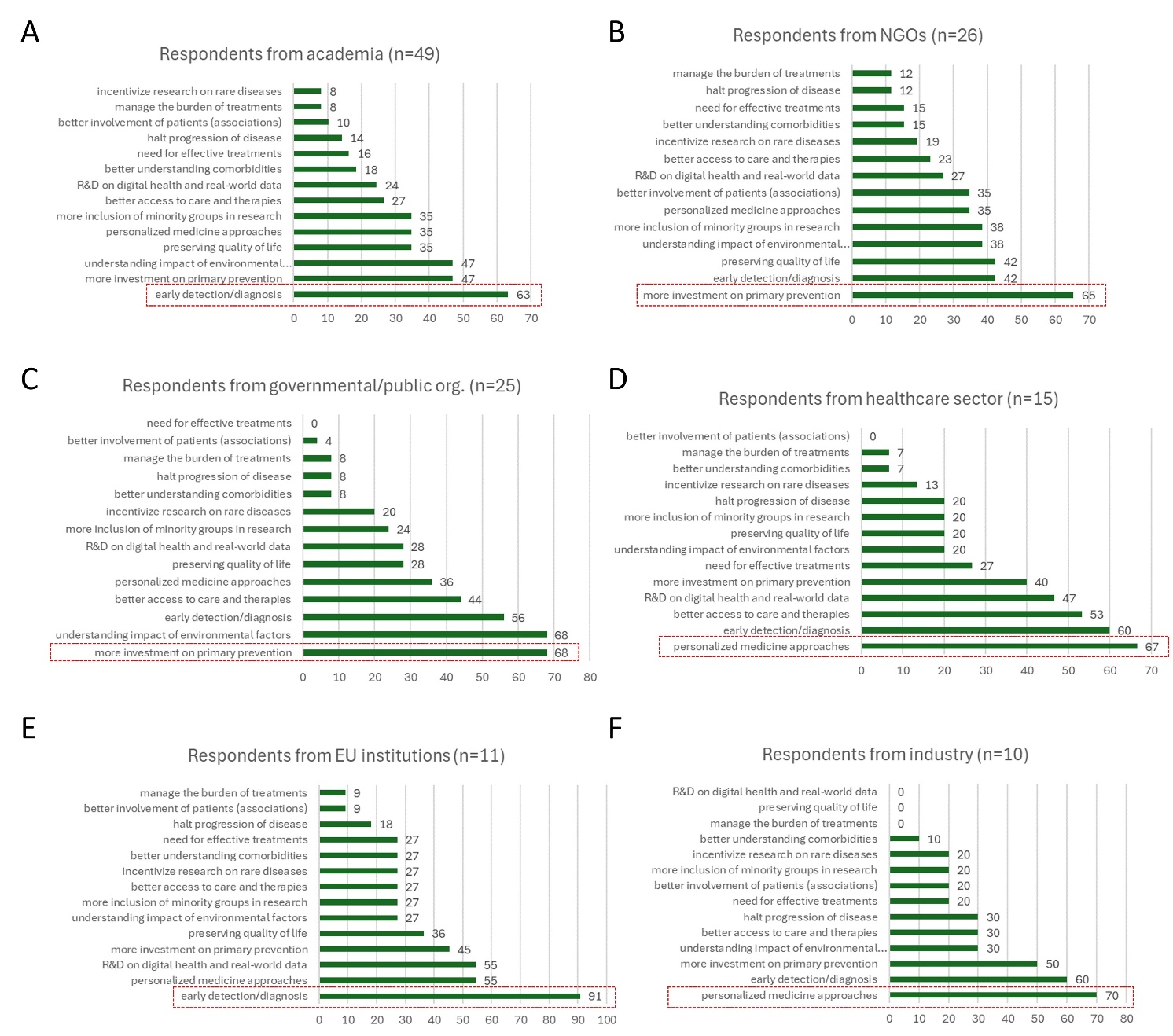


**Supplementary Figure 2.** Unmet medical needs’ ranking across different stakeholder groups: (A) academia, (B) NGOs, (C) government/public organisations, (D) healthcare, (E) EU institutions, and (F) industry. Participants were allowed to select between 1 and 5 options, which explains why the combined percentages exceed 100%.


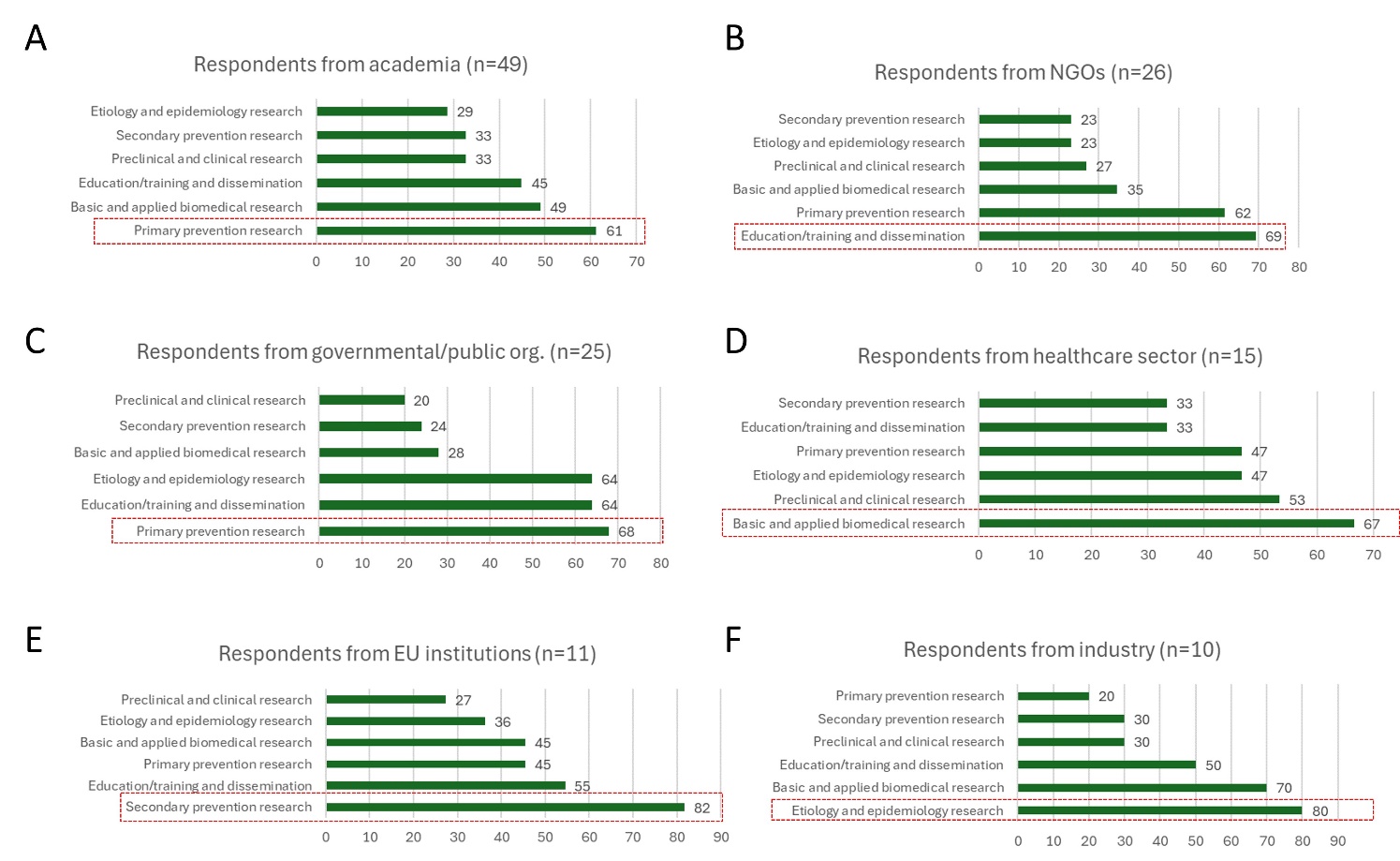


**Supplementary Figure 3.** Ranking of research areas deserving more funding across different stakeholder groups: (A) academia, (B) NGOs, (C) government/public organisations, (D) healthcare, (E) EU institutions, and (F) industry. Participants were allowed to select between 1 and 3 options options, which explains why the combined percentages exceed 100%.


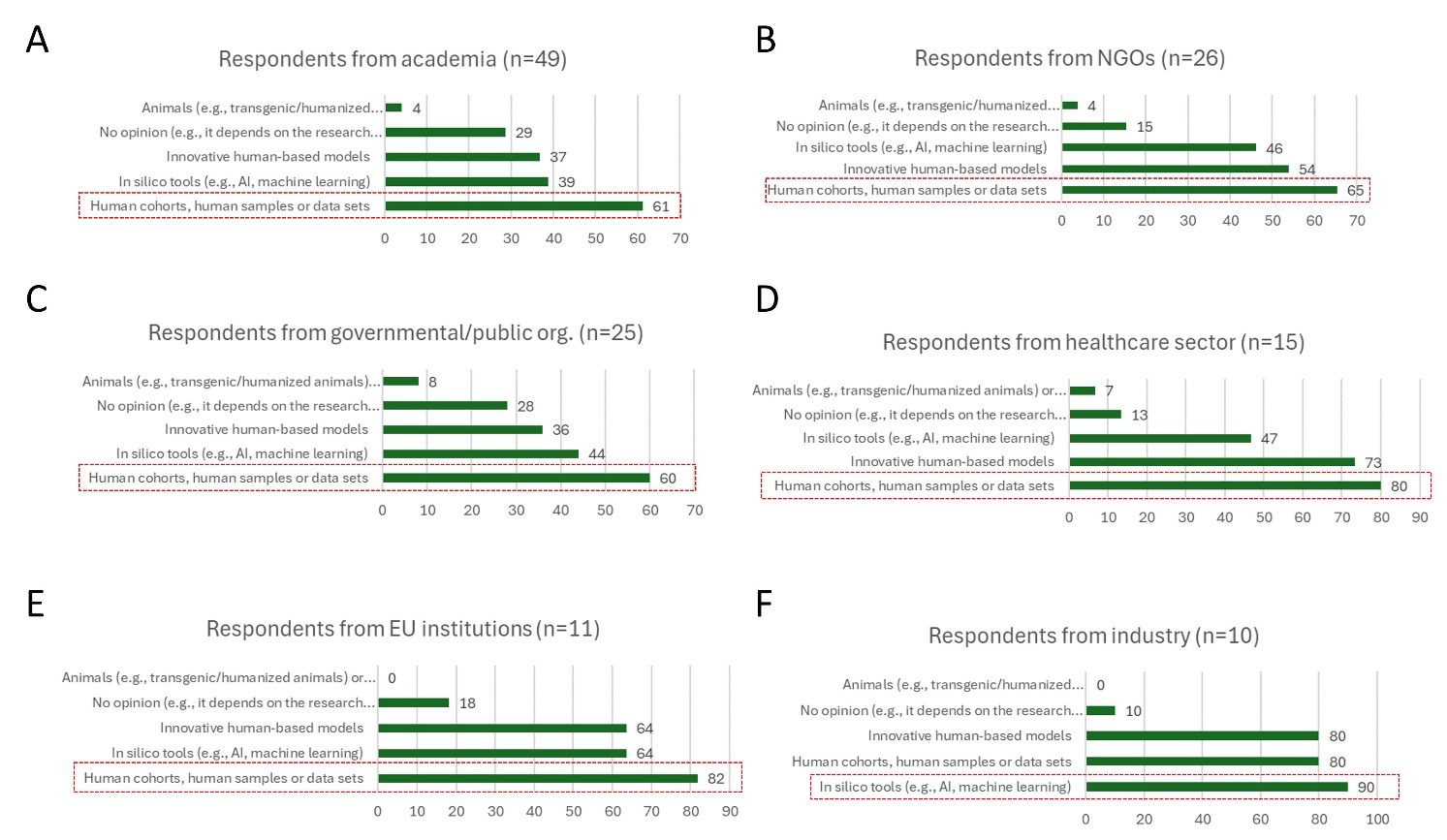


**Supplementary Figure 4.** Ranking of research approaches considered most conducive to impact across different stakeholder groups: (A) academia, (B) NGOs, (C) government/public organisations, (D) healthcare, (E) EU institutions, and (F) industry. Participants were allowed to select more than one option, which explains why the combined percentages exceed 100%.
